# Transient, flexible gene editing in zebrafish neutrophils and macrophages for determination of cell-autonomous functions

**DOI:** 10.1242/dmm.047431

**Published:** 2021-07-23

**Authors:** Abdulsalam I. Isiaku, Zuobing Zhang, Vahid Pazhakh, Harriet R. Manley, Ella R. Thompson, Lucy C. Fox, Satwica Yerneni, Piers Blombery, Graham J. Lieschke

**Affiliations:** 1 Australian Regenerative Medicine Institute, Monash University, Clayton, VIC 3800, Australia; 2 Department of Biological Sciences, School of Life Science, Shanxi University, Taiyuan, Shanxi Province 030006, China; 3 Department of Pathology, Peter MacCallum Cancer Centre, Melbourne, VIC 3000, Australia; 4 Sir Peter MacCallum Department of Oncology, The University of Melbourne, Parkville, VIC 3010, Australia; 5 Department of Clinical Haematology, Peter MacCallum Cancer Centre and The Royal Melbourne Hospital, Parkville, VIC 3050, Australia

**Keywords:** Cell autonomy, CRISPR-Cas9, Gene editing, Macrophages, Neutrophils, Zebrafish

## Abstract

Zebrafish are an important model for studying phagocyte function, but rigorous experimental systems to distinguish whether phagocyte-dependent effects are neutrophil or macrophage specific have been lacking. We have developed and validated transgenic lines that enable superior demonstration of cell-autonomous neutrophil and macrophage genetic requirements. We coupled well-characterized neutrophil- and macrophage-specific Gal4 driver lines with *UAS:Cas9* transgenes for selective expression of Cas9 in either neutrophils or macrophages. Efficient gene editing, confirmed by both Sanger and next-generation sequencing, occurred in both lineages following microinjection of efficacious synthetic guide RNAs into zebrafish embryos. In proof-of-principle experiments, we demonstrated molecular and/or functional evidence of on-target gene editing for several genes (*mCherry*, *lamin B receptor*, *trim33*) in either neutrophils or macrophages as intended. These new *UAS:Cas9* tools provide an improved resource for assessing individual contributions of neutrophil- and macrophage-expressed genes to the many physiological processes and diseases modelled in zebrafish. Furthermore, this gene-editing functionality can be exploited in any cell lineage for which a lineage-specific Gal4 driver is available.

This article has an associated First Person interview with the first author of the paper.

## Abstract (precis)

**Summary:** We developed new tools for lineage-specific gene editing in neutrophils or macrophages based on leukocyte-specific Cas9 expression, that can be used with injected synthetic gRNAs.

## INTRODUCTION

Neutrophils and macrophages, despite both being phagocytes, have many lineage-specific functions and contribute differentially to physiological and pathological processes. Zebrafish models have made important contributions to separating distinctive roles of the two phagocytes, including during infection (Hosseini et al., 2016), inflammation (Loynes et al., 2018) and regeneration (Pase et al., 2012); however, the available tools to segregate phagocyte roles have limitations.

Transplantation is feasible, but technically challenging in embryonic/larval systems (Pase et al., 2012). Conditional lineage-specific neutrophil or macrophage ablation by metronidazole/nitroreductase systems reduces numbers of the individual phagocyte type, but by acutely invoking apoptosis, which itself may not be physiologically neutral (Ellett et al., 2018; Okuda et al., 2015; Prajsnar et al., 2012). Transcriptional factor manipulations, such as *irf8* knockdown, can reduce numbers of macrophages, but at the same time increase the abundance of neutrophils, so macrophage requirement is not tested in the absence of any effect on neutrophils (Li et al., 2011; Rhodes et al., 2005). Lineage-specific requirements can be assigned to genes with lineage-specific expression, but this dichotomy only rarely applies and is reliant on the independent marker(s) used to assign leukocyte lineage identity.

Lineage-specific gene editing would provide the opportunity to assess autonomous functional requirements, by selective knockdown of a gene of interest in one cell type. Several configurations have been successfully used in zebrafish (Ablain et al., 2015; Di Donato et al., 2016; Dong et al., 2009; Yin et al., 2015; Zhou et al., 2018; Wang et al., 2021). These include a neutrophil-specific gene editing based on *lyzC* promoter transgenes that directly drive nuclear-localized Cas9 and guide RNA (gRNA) expression (Zhou et al., 2018) and a recent refinement of this system (Wang et al., 2021).

We report an improved, validated system for lineage-specific phagocyte gene editing in zebrafish. By employing our characterized, lineage-specific, Gal4-based neutrophil and macrophage drivers to express *UAS:Cas9* transgenes, we sought to achieve augmented levels of Cas9 expression in each leukocyte lineage (Ellett et al., 2011; Okuda et al., 2015). By developing the system for gene editing in either neutrophils or macrophages in parallel, we provide, for the first time, the opportunity to experimentally segregate the functional requirement for genes expressed in both phagocyte types to one or other of these individual phagocyte lineages. This provides a new, tractable, experimental opportunity to better define neutrophil- and macrophage-specific cell-autonomous gene functionality in the full range of zebrafish physiological and disease models. Furthermore, as the well-validated *UAS:Cas9* transgenes can be coupled with any of the numerous other lineage-specific Gal4 lines available by an appropriate breeding strategy, the gene-editing functionality can be exploited in any cell lineage for which a lineage-specific Gal4 driver is available.

## RESULTS

### Generation of transgenic zebrafish expressing Cas9 in either neutrophils or macrophages

**Fig. 1.**
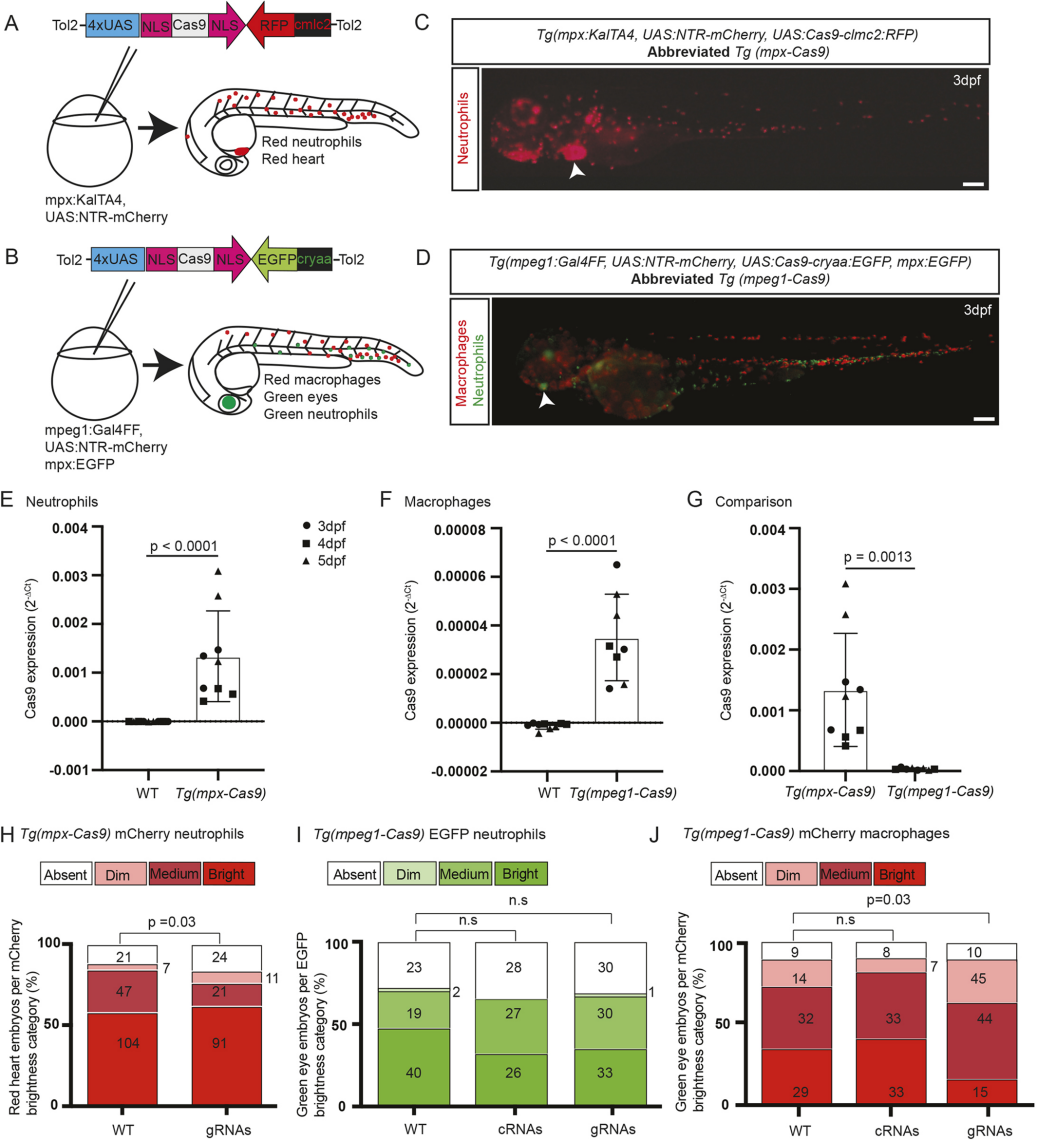
**Transgenes for leukocyte lineage-specific gene editing in zebrafish.** (A) For gene editing in neutrophils, a *tol2*-flanked *UAS:Cas9* construct was microinjected into one-cell-stage *Tg(mpx:KalTA4)* embryos. Nuclear localization signal (NLS) motifs target Cas9 to the nucleus. This construct carried a *Tg(clmc2-RFP)* tracer marker. (B) For gene editing in macrophages, a different *tol2*-flanked *UAS:Cas9* construct was microinjected into one-cell-stage *Tg(mpeg1:Gal4FF)* embryos that carried a *Tg(cryaa-EGFP)* tracer marker. Each background carried a *Tg(UAS:NTR-mCherry)* transgene. (C,D) Embryos with red leukocytes from the *Tg(UAS:NTR-mCherry)* reporter driven by either *mpx:KalTA4* (neutrophil) or *mpeg1:Gal4FF* (macrophage) transgenes, co-segregating with the two different *UAS:Cas9* transgenes indicated by their respective tracer marker [white arrowheads, red heart (C) and green eye (D)], shown for F2 *Tg(mpx-Cas9)* (C) and F3 *Tg(mpeg1-Cas9)* (D) embryos. The *Tg(mpeg1-Cas9)* line also carried *Tg(mpx:EGFP)*, marking neutrophils green (D). Scale bars: 200 µm. (E-G) Quantitative PCR showing relative Cas9 mRNA expression for *Tg(mpx-Cas9)* (E), *Tg (mpeg-Cas9)* (F) and a comparison of both (G). Data normalized to housekeeping gene (*ppial*) and represent mean replicates of three runs at different time points differentiated by shapes: black circles, 3 dpf; black squares, 4 dpf; black triangles, 5 dpf. No differences exist between timepoints within genotypes. Unpaired two-tailed Student’s *t*-test. *P*-values are shown. (H-J) mCherry reporter gene knockdown in *Tg(mpx-Cas9)* and *Tg(mpeg1-Cas9)* embryos following delivery of a multiplexed mCherry gRNA pair targeting the transcript from the *UAS:NTR-mCherry* reporter (gRNA validation presented in Fig. S3). Embryos for gRNA injection were generated from *Tg(mpx-Cas9)* and *Tg(mpeg1-Cas9)* incrosses. As the incrossed fish were not confirmed as homozygous for all transgenes, there was the possibility of segregation of the Gal4 driver, *UAS:Cas9* effector and *UAS:NTR-mCherry* reporter transgenes; hence, a categorical scoring system (absent/dim/medium/bright) was used to detect a shift towards reduced mCherry expression (further details in Fig. S3). Prior to scoring, embryos were selected for the independent backbone markers confirming the presence of the *UAS:Cas9* effector [red heart for *Tg(mpx-Cas9)* (C, white arrowhead); green eye for *Tg(mpeg1-Cas9)* (D, white arrowhead)]; this line also carries the *Tg(mpx:EGFP)* marker resulting in green neutrophils. (H) Knockdown of mCherry expression in *Tg(mpx-Cas9)* neutrophils [percentage absent+dim, 15.6% (WT) versus 23.8% (gRNA injected)]. (I,J) A similar shift towards duller categories occurred in *Tg(mpeg1-Cas9)* mCherry macrophages (J), without any alteration in the distribution of EGFP-expressing neutrophils, serving as an internal negative control (I). Pooled data from two (H) and three (I,J) independent experiments, total pooled *n* values are shown within columns. *P*-values from chi-square test. n.s., not significant.

To generate a leukocyte lineage-specific gene-editing system in zebrafish, we expressed Cas9 in either neutrophils or macrophages by taking advantage of our established, well-characterized neutrophil and macrophage Gal4 driver lines: for neutrophils, *Tg(mpx:KalTA4)* (Okuda et al., 2015); and for macrophages, *Tg(mpeg1:Gal4FF)* (Ellett et al., 2011). Zebrafish codon-optimized *Streptococcus pyogenes* Cas9 complementary DNA (cDNA) was flanked by two nuclear localization signal (NLS) sequences to localize Cas9 protein to the nucleoplasm (Fig. 1A,B) (Jao et al., 2013). Two *tol2*-flanked *UAS-Cas9* transgenes were built in plasmid backbones incorporating either *cmlc2-RFP* or *cryaa-EGFP* markers, to enable phenotypic tracking of the Cas9 transgene independent of its expression of Cas9 protein in leukocytes.

The *cmlc2-RFP* or *cryaa-EGFP* transgenes were microinjected into the neutrophil and macrophage driver lines, respectively. As the injected Gal4 driver lines also carried the *UAS:NTR-mCherry* transgene (Davison et al., 2007), this transgene configuration resulted in lines with mCherry-expressing phagocytes in which, by virtue of the breeding strategy, a red heart indicated Cas9 expression in mCherry neutrophils, and a green eye indicated Cas9 expression in mCherry macrophages (Fig. 1C,D). Additionally, the *Tg(mpeg1:Gal4FF, UAS:NTR-mCherry, UAS:Cas9)* line carried the *mpx:EGFP* transgene (Renshaw et al., 2006), marking neutrophils green. Stable transgenic F1 zebrafish embryos expressing the red heart and green eye fluorophore markers were confirmed as carrying the linked Cas9 transgene by polymerase chain reaction (PCR) genotyping (Fig. S1). Table 1 presents full details of the compound transgenic lines used in these studies and the backbone markers used to facilitate genetic selection. For simplicity, these compound transgenic lines are hereafter called *Tg(mpx-Cas9)*, for Cas9 expression in neutrophils, and *Tg(mpeg1-Cas9)*, for Cas9 expression in macrophages.

**Table 1.**
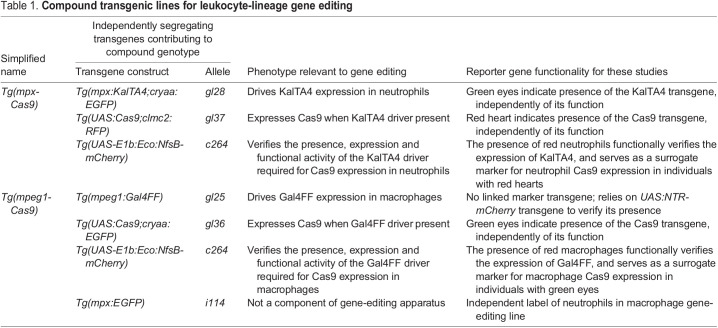
Compound transgenic lines for leukocyte-lineage gene editing

### Cas9 transgene expression in zebrafish phagocytes

Transgene expression was examined qualitatively by reverse-transcription PCR (RT-PCR) of RNA extracted either from pooled embryos or fluorescence-activated cell sorting (FACS)-purified adult neutrophils or embryonic macrophages, and examining the PCR products by gel electrophoresis (Fig. S2). Cas9 mRNA expression was detected in *Tg(mpx-Cas9)* embryos and in adult neutrophils from whole-kidney marrow (WKM) of this line purified based on *mpx*-driven mCherry reporter gene expression, and in *Tg(mpeg1-Cas9)* embryos and purified embryonic macrophages. Cas9 reverse-transcription quantitative PCR (RT-qPCR) expression analysis in whole embryos also detected Cas9 transgene expression in *Tg(mpx-Cas9)* and *Tg(mpeg1-Cas9)* embryos but not in wild-type (WT) embryos (Fig. 1E,F), and showed that there was significantly more Cas9 expression in *Tg(mpx-Cas9)* embryos than in *Tg(mpeg1-Cas9)* embryos (Fig. 1G). This 38-fold difference in Cas9 expression levels between *Tg(mpx-Cas9)* and *Tg(mpeg1-Cas9)* embryos is a cumulative outcome of the relative abundance of the two leukocyte cell types, differential promoter strengths and the different Gal4 variant coupled to each.

### Functional gene editing from Cas9 transgene expression in zebrafish phagocytes

Although gRNAs are customarily injected complexed to Cas9 because this is believed to enhance their stability (Cong et al., 2013; Gagnon et al., 2014; Hwang et al., 2013; Jinek et al., 2012), we tested whether microinjected gRNAs injected directly into Cas9-expressing embryos would result in gene editing. We used commercially synthesized gRNAs incorporating proprietary modifications to stabilize them.

To test whether gene editing with functional outcomes was achieved by phagocyte-expressed Cas9, we initially used mCherry gRNAs, hypothesizing that the mCherry reporter, driven by the same Gal4/UAS couple, would be expressed at the right time to be susceptible to Cas9 gene editing in the presence of gRNAs. Multiplexed delivery of two mCherry gRNAs, validated for gene-editing activity in a T7E1 assay (Fig. S3), significantly increased the proportion of *Tg(mpx-Cas9)* and *Tg(mpeg1-Cas9)* embryos with no or dimmer populations of mCherry-expressing cells (Fig. 1H-J). Although this outcome is phenotypically consistent with on-target mCherry gene editing in neutrophils and macrophages, respectively, Sanger sequencing of FACS-purified mCherry-positive cells from these two scenarios did not show convincing evidence of sequence heterogeneity across the genomic DNA target sites. We interpret this as resulting from an anticipated bias against finding on-target gene editing in cells that retained mCherry expression, because the most efficaciously edited phagocytes would not express any fluorophore and hence be excluded from the sorted cell population.

**Fig. 2.**
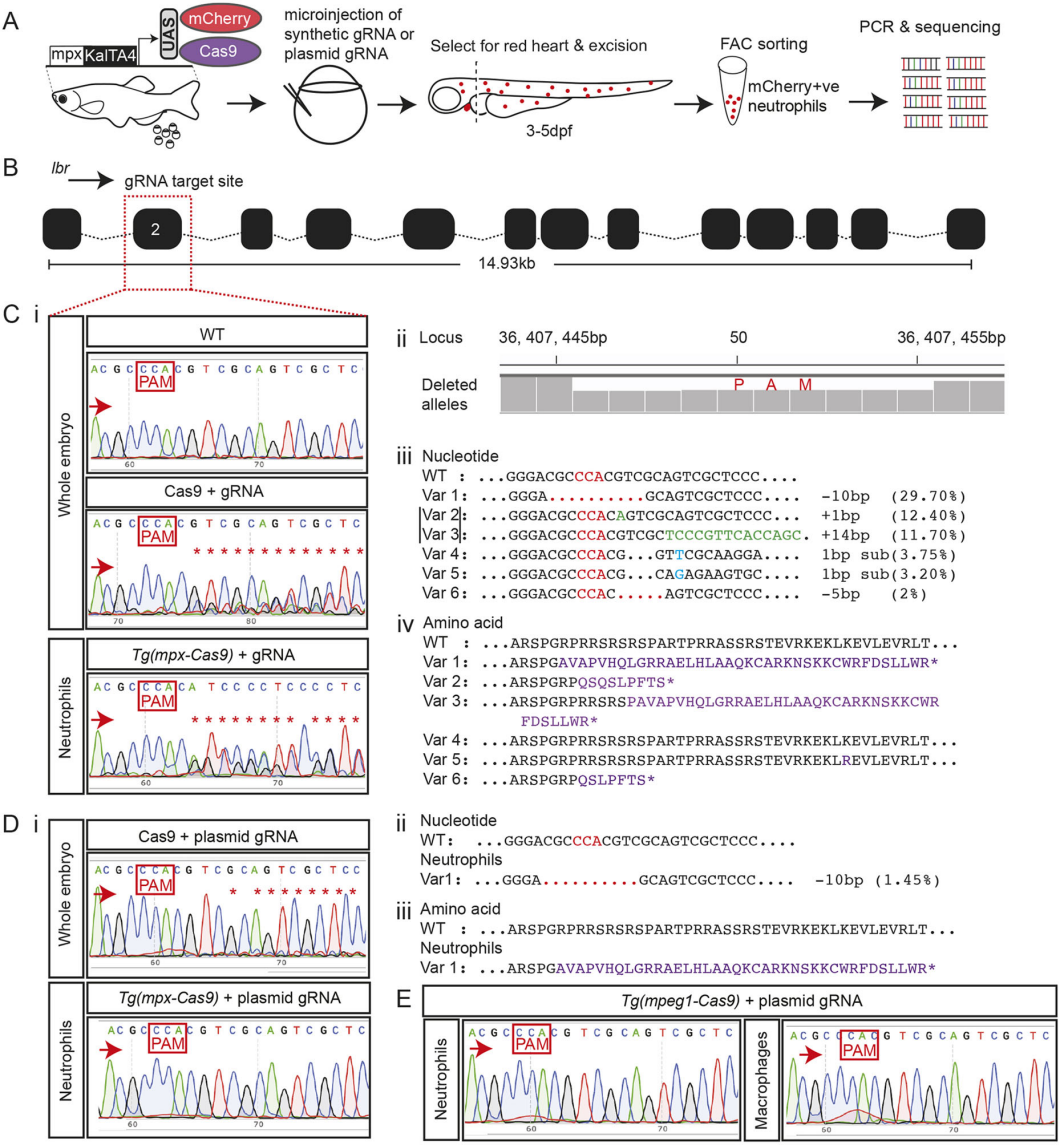
**On-target *lamin B receptor* (*lbr*) gene editing in neutrophils of *Tg(mpx-cas9*) zebrafish embryos.** (A) Schematic of experimental steps for *in vivo* gene editing in neutrophils. (B) Zebrafish *lbr* locus showing gRNA target site in exon 2. (C) High-level on-target *lbr* gene editing from synthetic gRNA delivery. (Ci) Sanger sequencing chromatogram of wild-type (WT) whole-embryo DNA (upper panel; non-edited control) compared to that from F3 embryos injected with synthetic *lbr* gRNA complexed to exogenous Cas9 protein (middle panel; positive control) and neutrophil-lineage gene editing in fluorescence-activated cell sorting (FACS)-purified neutrophils from *Tg(mpx-Cas9)* embryos injected with synthetic *lbr* gRNA (lower panel). (Cii) Manhattan plot from next-generation sequencing (NGS) of the same neutrophil DNA preparation as Ci (lower panel), displaying cumulative distribution of aligned deleted alleles at the target locus. (Ciii) NGS of the same DNA preparation as Ci (lower panel) revealed six predominant variants (Var 1-6, bracketed Var 2 and 3 occurred in *cis*), representing 62.75% on-target gene editing. None of these variants was seen in DNA from embryos not injected with *lbr* gRNA. (Civ) Predicted amino acid sequences of variants 1-6. A high proportion of gene edits (70.28%), representing 44.1% of the NGS reads, are predicted to be nonsense mutations. (D) Low-level on-target *lbr* gene editing from plasmid gRNA delivery using a plasmid encoding *lbr* gRNA expressed from the U6 promoter (plasmid gRNA). (Di) Upper panel shows Sanger sequencing chromatogram of DNA from whole 3 dpf embryos injected with 1.5 ng/µl plasmid gRNA and exogenous Cas9 enzyme, serving as a positive control, showing low-level gene editing from plasmid gRNA delivery when Cas9 is in abundance. Lower panel shows Sanger sequencing chromatogram of DNA from FACS-purified neutrophils of 5 dpf *Tg(mpx-Cas9)* embryos injected with 30 ng/µl *lbr* plasmid gRNA alone, showing no detectable gene editing. (Dii) NGS of the same neutrophil DNA preparation as in Di (lower panel) detected one gene-edited variant (Var 1), representing 1.45% on-target gene editing. No gene editing was detected by NGS in ‘other cells’ from these same embryos. This variant was not seen in DNA from embryos not injected with *lbr* plasmid gRNA. (Diii) Predicted amino acid sequence of variant 1 results in a nonsense mutation. (E) No gene editing was detected by Sanger sequencing in FACS-purified macrophages from 3 dpf *Tg(mpeg1:Cas9)* embryos (right panel). FACS-purified neutrophils serve as an internal negative control (left panel). PAM, protospacer adjacent motif highlighted in red boxes and red font; red arrows indicate the sequencing direction; red asterisks mark sequence heterogeneity due to on-target gene editing; red dots indicate deletion; blue font, substituted nucleotides; green font, inserted nucleotides; purple font, truncated protein; black dots, sequence continues as WT.

We therefore assessed gene editing molecularly in an alternative scenario (Fig. 2A), independent of the leukocyte reporter fluorophore and at a locus that did not affect neutrophil abundance. We chose a highly efficient gRNA previously used successfully to target the zebrafish *lamin B receptor* (*lbr*) gene (Fig. 2B) (Manley, 2019). gRNA activity following co-injection of the *lbr* gRNA with exogenous Cas9 protein was confirmed as detectable by Sanger chromatogram sequence heterogeneity in whole embryos (Fig. 2Ci). FACS-purified neutrophils from the *lbr* gRNA-microinjected *Tg(mpx-Cas9)* zebrafish embryos also showed evidence of on-target gene editing by Sanger sequencing of genomic DNA, suggesting ∼50% prevalence of gene modification (Fig. 2Ci). NGS of this same FACS-purified neutrophil genomic DNA demonstrated six predominant variants representing a 62.75% editing frequency (Dataset 1). Four variants accounting for ∼44.1% of NGS reads are predicted to result in premature stop codon and hence loss of protein function (Fig. 2Ciii,iv). These variants were not represented in NGS-sequenced WT zebrafish genomic DNA prepared similarly.

In an attempt to achieve more sustained delivery of the gRNA to maintain lineage-specific gene editing during ongoing leukocyte development, we inserted the *lbr* gRNA-encoding sequence into a modified *tol2*-flanked plasmid-based transfer RNA (tRNA) delivery system (Shiraki and Kawakami, 2018). When this *lbr* gRNA plasmid was injected into WT embryos with Cas9 enzyme, low-level gene editing was detectable by Sanger sequencing, confirming that the plasmid-based delivery system was functional (Fig. 2Di, upper panel). This *lbr* gRNA delivery plasmid was microinjected into one-cell-stage embryos from both *Tg(mpx-Cas9)* and *Tg(mpeg1-Cas9)*, and the extent of on-target gene editing achieved in neutrophils and macrophages was assessed in FACS-purified populations from 3-5 days post-fertilization (dpf) embryos (Fig. 2D,E). Although gene editing occurred in *Tg(mpx-cas9)* neutrophils, the proportion of gene-edited transcripts was low and only detected by NGS (1.45% of transcripts, mean of two NGS replicates; Dataset 1) (Fig. 2Dii,iii). This was far less gene editing than the 62.75% in neutrophils from 3 dpf *Tg(mpx-Cas9)* embryos after microinjection of synthetic *lbr* gRNA (Fig. 2Ciii). No gene editing in *Tg(mpeg1-Cas9)* macrophages was detected by Sanger sequencing following plasmid *lbr* gDNA delivery (Fig. 2E).

Although the incidence of neutrophil-lineage gene editing in this experiment was low, the NGS data permitted on-target neutrophil lineage specificity of *lbr* gene editing to be assessed quantitatively by comparing gene editing incidence in FACS-purified mCherry-labelled neutrophils compared with the mCherry-negative population of all other cell types from the same *Tg(mpx-Cas9)* embryos. One variant that shared similar incidences across control and test samples was regarded as a polymorphism (chr20:36407345G>A, synonymous). The gene-edited frameshift mutation chr20:36407446_36407455del frequency of 1.45% in neutrophil DNA was not found in other cells from the same embryos.

Collectively, these data provide evidence of on-target gene editing in both phagocyte *Tg(mpx-Cas9)* and *Tg(mpeg1-Cas9)* lines, and comprehensive molecular evidence for gene editing with neutrophil-lineage specificity in the *Tg(mpx-Cas9)* system, and demonstrate that synthetic gRNA is more effective than transient plasmid-based gRNA delivery for transient gene editing at these early developmental time points.

### Demonstration of utility: assessment of cell autonomy of the *trim33* requirement for macrophage and neutrophil migration

In the germline *trim33* mutant *moonshine*, neutrophils and macrophages develop in normal abundance, but display profound migration defects in response to inflammatory cues (Demy et al., 2017). In this mutant, the neutrophil migration defect was demonstrated to be cell autonomous by transplantation, although a similar experiment was not performed for macrophages. This scenario provided an experimental model for using *Tg(mpx-Cas9)* and *Tg(mpeg1-Cas9)* lines to test for the experimentally proven and presumed cell-autonomous *trim33* requirement in neutrophil and macrophage migration, respectively.

**Fig. 3.**
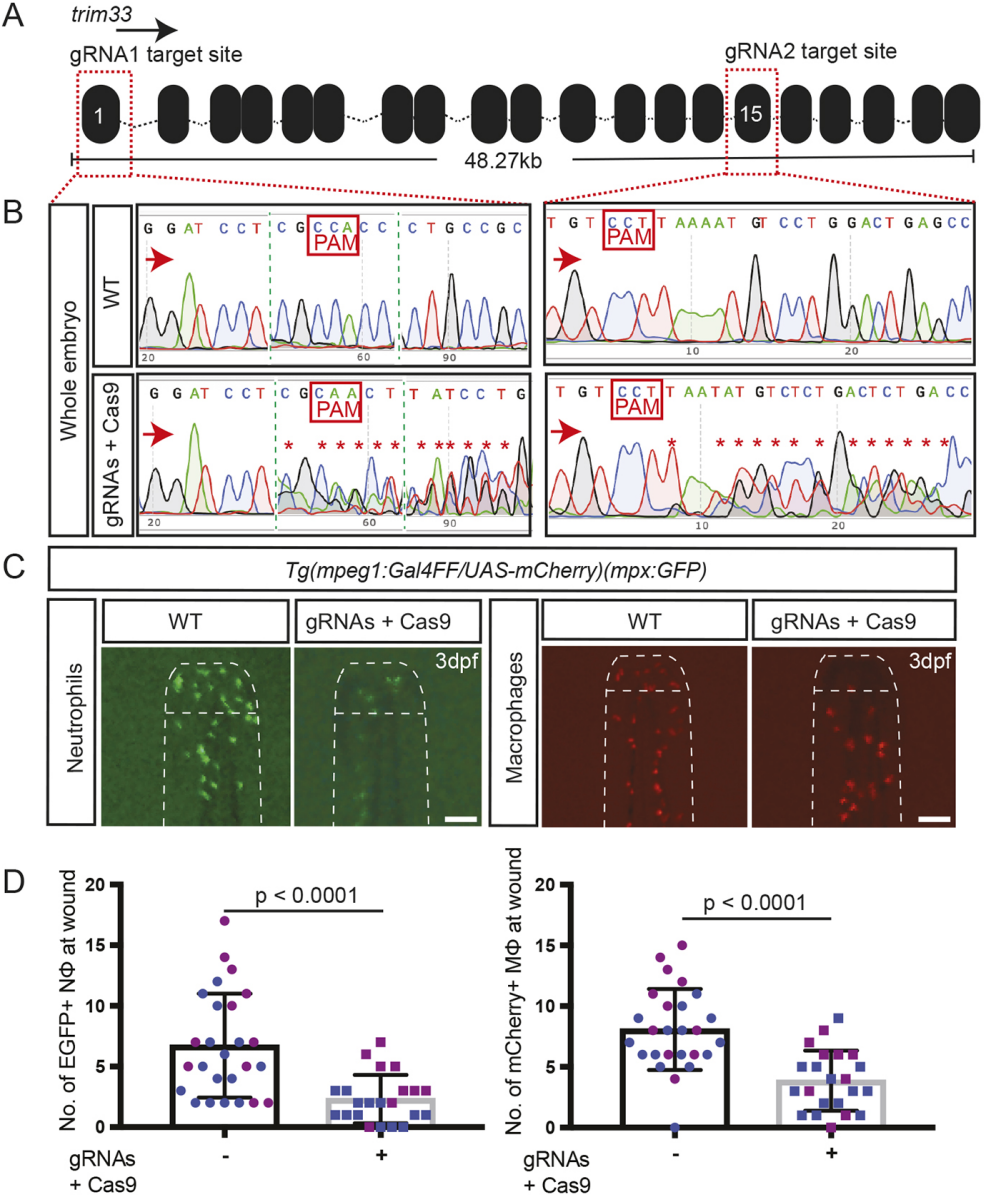
**Whole-body *trim33* crispants phenocopy the neutrophil and macrophage migration defects of *moonshine*/*trim33* mutants.** (A) Zebrafish *trim33* locus showing target sites for two gRNAs in exon 1 and 15. (B) Sanger chromatogram of WT whole-embryo DNA (upper row) compared to F0 *Tg(mpeg1:Gal4FF/UAS:NTR-mCherry)(mpx:GFP)* crispant embryos injected with two multiplexed *trim33* gRNAs complexed to exogenous Cas9 protein (lower row). (C) Fluorescent images of GFP-labelled neutrophils and mCherry-labelled macrophages at 3.5 h after caudal fin transection, in WT and *trim33* crispant 3 dpf *Tg(mpeg1:Gal4FF/UAS:NTR-mCherry)(mpx:GFP)* embryos. Embryos from the same experiment as in B. (D) Neutrophil and macrophage numbers at wound site at 3.5 h post-injury. Red arrows indicate the sequencing direction; red asterisks indicate sequence heterogeneity due to on-target gene editing; green vertical dashed lines indicate cropped areas of the chromatogram; PAM, protospacer adjacent motif highlighted in red boxes. Unpaired two-tailed Student’s *t*-test (*P*<0.0001) of pooled data from two independent experiments indicated by different colours. Scale bars: 100 µm.

First, the effects of indiscriminate whole-embryo *trim33* gene editing were evaluated, which we hypothesized should replicate the *moonshine* phenotype. Two *trim33* gRNAs (gRNA1 and gRNA2) were validated for on-target functional gene-editing efficacy by co-injecting them together complexed with exogenous Cas9, into *Tg(mpeg1:Gal4FF, UAS:NTR-mCherry, mpx:EGFP*) triple-transgenic reporter lines, which have green neutrophils and red macrophages. Sanger sequencing of whole-embryo DNA across both gRNA-targeted protospacer adjacent motif (PAM) sites showed highly heterogeneous chromatograms, indicating very efficacious gene editing with both these gRNAs (Fig. 3A,B). At the high level of gene editing demonstrated in this Sanger chromatograms, these F0 ‘crispant’ embryos replicated the migration defect of *moonshine* mutants, with reduced numbers of migrating neutrophils and macrophages present at the wound at 3.5 h post-injury (Fig. 3C,D). We also scored leukocyte abundance by counting the total number of trunk neutrophils distal to the yolk sac tip. In contrast to the previous report of normal neutrophil abundance in *trim33* mutants (Demy et al., 2017), we observed a 19.6% reduction in total trunk neutrophil number in global crispants (Fig. S4Ai), but when we adjusted for this, a proportional neutrophil migration defect was still evident (Fig. S4Aii). The total number of trunk macrophages in these crispants was normal (Fig. S4Aiii). We did not explicitly examine for other phenotypic features of the *moonshine* mutant [such as anaemia (Ransom et al., 1996) and lack of microglia (Demy et al., 2017)] in the global crispants.

**Fig. 4.**
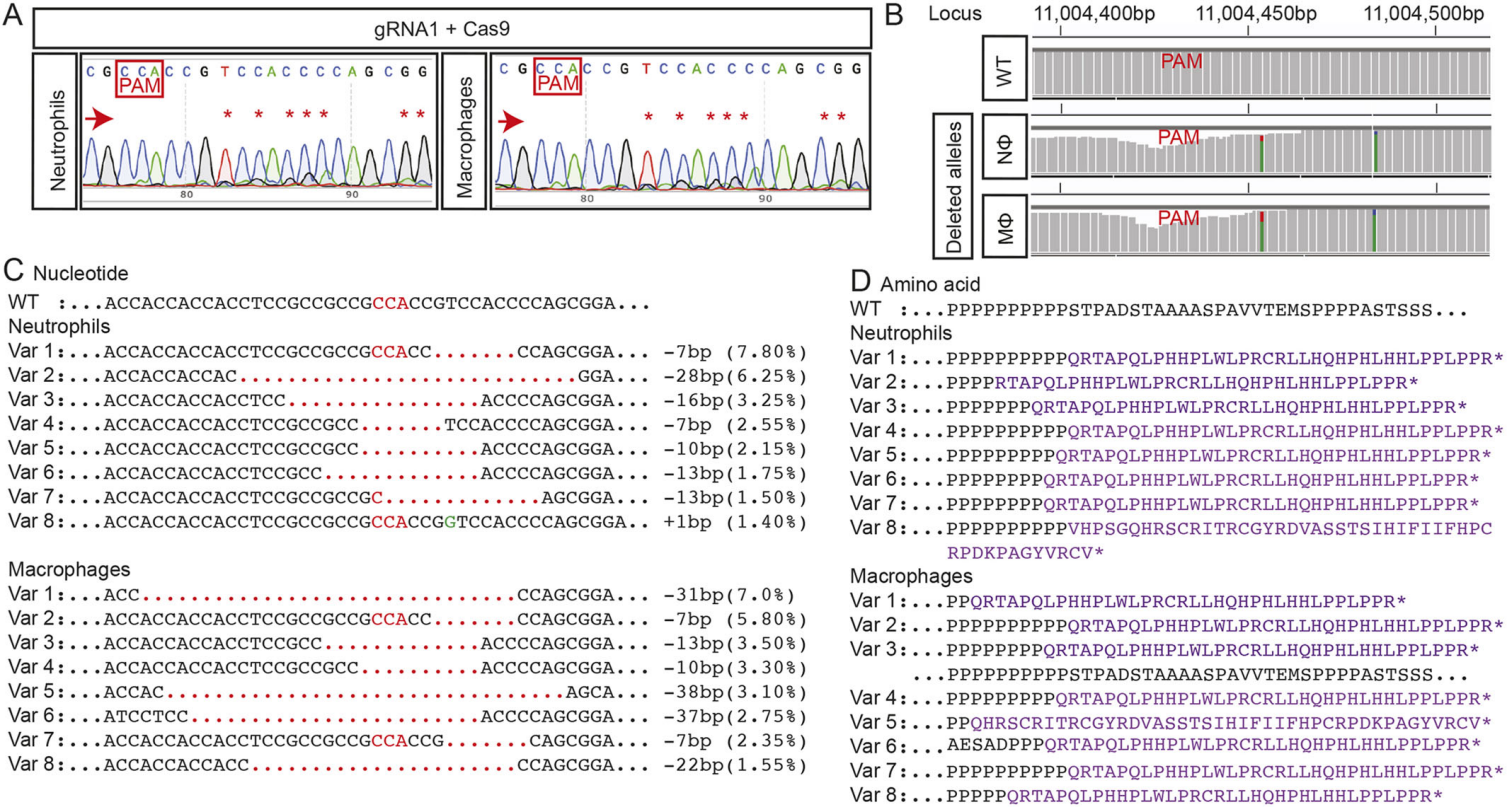
**Neutrophils and macrophages from whole-body *trim33* crispants have on-target gene editing.** (A) Sanger chromatograms of DNA from FACS-purified neutrophils and macrophages from whole-body 3.5 dpf *Tg(mpeg1:Gal4FF/UAS:NTR-mCherry)(mpx:GFP)* crispant embryos injected with *trim33* gRNA1 complexed with Cas9, demonstrating on-target gene editing in both leukocyte types. (B) Manhattan plots from NGS of the same DNA preparation as in A, displaying cumulative distribution of aligned deleted alleles at the target locus in neutrophils and macrophages. (C) There is a single allele in WT and eight predominant variants (Var 1-8), representing on-target gene-editing rates of 26.65% in neutrophils and 29.35% in macrophages. (D) Predicted amino acid sequences of variants 1-8; all are predicted to be nonsense mutations. Red arrows indicate the sequencing direction; red asterisks mark sequence heterogeneity; PAM, protospacer adjacent motif highlighted in red boxes and font; red dots, deletion; green font, insertion; purple font, truncated protein; black dots, sequence continues as WT.

The molecular nature of *trim33* gene editing achieved in the leukocytes of these crispant embryos was independently determined in a separate experiment focusing on gRNA1 targeting, in which FACS-purified populations of EGFP-positive neutrophils and mCherry-positive macrophages from embryos were similarly injected with just gRNA1 and exogenous Cas9 complexes. For both leukocyte types, on-target gene editing was evident in Sanger chromatograms across the gRNA1 PAM target site (Fig. 4A), and also in NGS analysis of the same samples (Fig. 4B,C). This confirms, at a molecular level, that gRNA1-targeted gene editing occurred at 26.65% and 29.35% efficiency, which would have the predicted outcome of truncating translated Trim33 protein in neutrophil and macrophages, respectively (Fig. 4D). It is important to note that this observation does not define the proportion of leukocytes in crispant embryos with at least one disrupted *trim33* locus when the two *trim33* gRNAs are injected together.

**Fig. 5.**
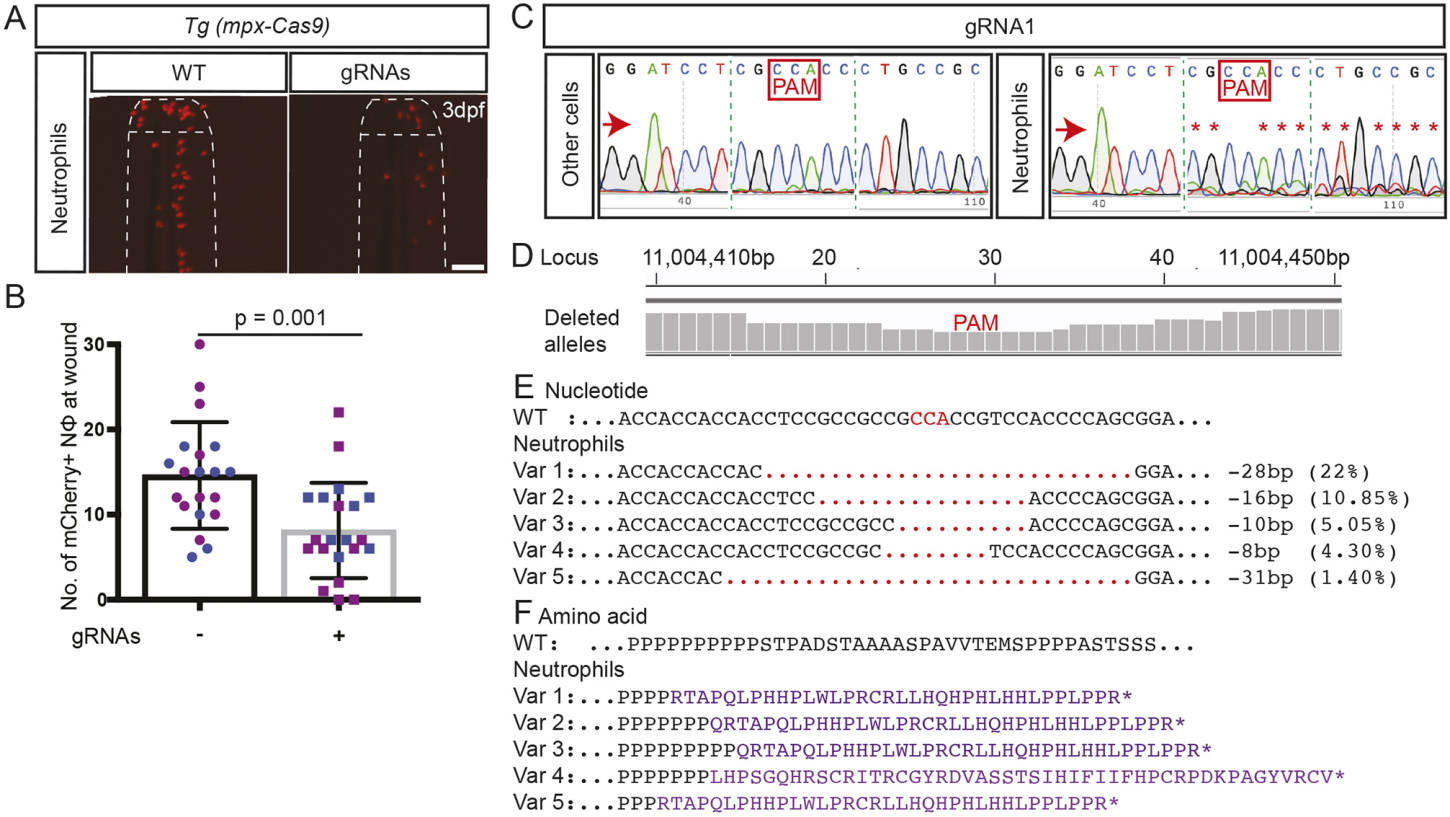
**Effects of *trim33* knockdown on neutrophil migration in *Tg(mpx-cas9*) zebrafish embryos.** (A) Representative fluorescent images of mCherry-labelled neutrophil at caudal fin 3.5 h post-wounding in 3 dpf *Tg(mpx-Cas9)* embryos injected with two *trim33* gRNAs, compared to uninjected transgenic embryos (WT), demonstrating the impaired migratory neutrophil response to caudal fin injury in gRNA-microinjected crispant embryos compared to WT. (B) Quantification of neutrophil response. (C) Sanger sequencing chromatogram of independent experiment demonstrating on-target Cas9 activity in FACS-purified mCherry-positive neutrophils. (D) Manhattan plots from NGS of the same neutrophil DNA preparation as in C, displaying cumulative distribution of aligned deleted alleles at the target locus. (E) WT reference sequence compared to five predominant variants (Var 1-5), representing 43.6% on-target gene editing in neutrophils. (F) Predicted amino acid sequences of variants 1-5 with nonsense mutations. Red arrows indicate the sequencing direction; red asterisks mark sequence heterogeneity; green vertical dashed lines indicate cropped areas of the chromatogram; PAM, protospacer adjacent motif highlighted in red boxes and font; red dots, deletion; purple font, truncated protein; black dots, sequence continues as WT. Unpaired two-tailed Student’s *t*-test (*P*=0.001) of pooled data from two independent experiments indicated by different colours. Scale bar: 100 µm.

Having validated these two gRNAs as efficacious at gene targeting when injected complexed with exogenous Cas9, we now tested their capacity to achieve ‘lineage-specific’ gene editing of functional consequence in the *Tg(mpx-Cas9)* and *Tg(mpeg1-Cas9)* lines. In the *Tg(mpx-Cas9)* system for neutrophil-lineage gene editing, the previously demonstrated cell-autonomous requirement in neutrophils for *trim33* was replicated (Fig. 5A,B). This phenotypic outcome correlated with Sanger sequencing showing highly effective on-target gene editing in neutrophils but not in other cells from the same embryos (Fig. 5C). NGS of purified neutrophils from these embryos demonstrated a maximal nucleotide deletion incidence of 43.6% at the PAM cut site (Fig. 5D,E), and all resulted in transcripts predicted to encode Trim33 proteins with early carboxyl truncations (Fig. 5F).

**Fig. 6.**
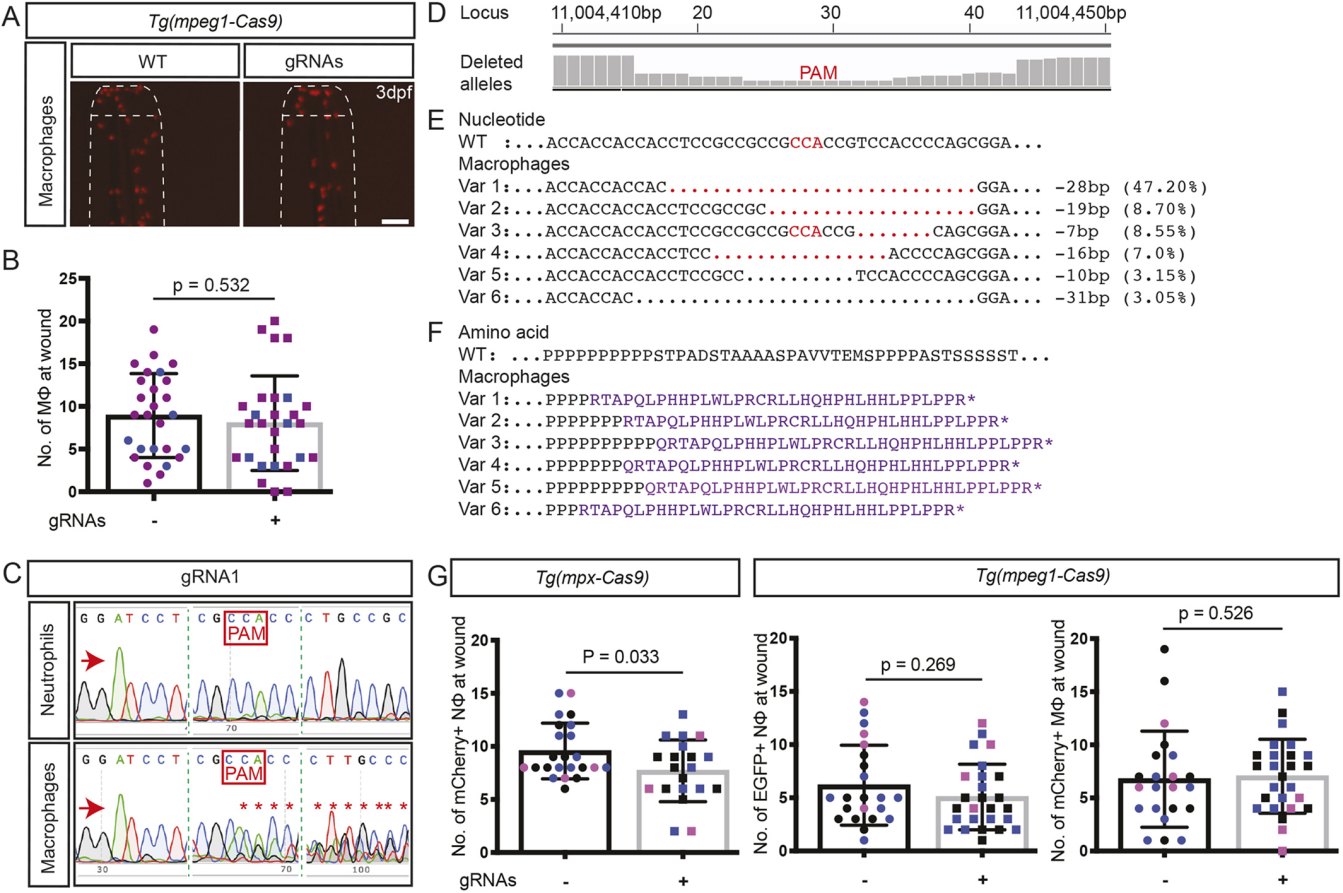
**On-target *trim33* gene editing in macrophages of *Tg(mpeg1-Cas9)* zebrafish embryos.** (A) Representative fluorescent images of mCherry-labelled macrophage at caudal fin 3.5 h post-wounding in 3 dpf *Tg(mpeg1-Cas9)* injected with two *trim33* gRNAs, compared to uninjected transgenic embryos (WT). (B) Quantification of macrophage migratory response. (C) Sanger sequencing chromatogram of an independent experiment, demonstrating on-target Cas9 activity in FACS-purified mCherry-positive macrophages. (D) Manhattan plots from NGS of the same DNA preparation as in C, displaying cumulative distribution of aligned deleted alleles at the target locus in macrophages. (E) WT reference sequence compared to six predominant variants (Var 1-6), representing 77.65% on-target gene editing in macrophages. (F) Predicted amino acid sequences of variants 1-6 with nonsense mutations. (G) Quantification of neutrophils at wound site in concurrent groups of *Tg(mpx-Cas9)* and *Tg(mpeg1-Cas9)* embryos injected with the same *trim33* gRNAs, showing a neutrophil migratory defect in *Tg(mpx-Cas9)* neutrophils but not in *Tg(mpeg1-Cas9)* neutrophils. The absence of a migration defect in *trim33* knockdown *Tg(mpeg1-Cas9)* macrophages (B) is replicated. Red arrows indicate the sequencing direction; red asterisks mark sequence heterogeneity; green vertical dashed lines indicate cropped areas of the chromatogram; PAM, protospacer adjacent motif highlighted in red boxes and font; red dots, deletion, purple font, truncated protein; black dots, sequence continues as WT. Unpaired two-tailed Student's *t*-test (*P*=0.53) of pooled data from two (B) and three (G) independent experiments indicated by different colours. Scale bar: 100 µm.

In contrast, despite very effective high-incidence gene editing within macrophages in the *Tg(mpeg1-Cas9)* line, a macrophage migration defect was not observed (Fig. 6A,B). Sanger chromatograms of DNA from FACS-purified macrophages displayed a level of sequence corruption at the PAM site consistent with at least 50% efficiency of gene editing (Fig. 6C). NGS of macrophage DNA demonstrated a maximal nucleotide deletion incidence of 84% at the PAM cut site (Fig. 6D). Six variants accounting for 77.65% of the NGS reads (Fig. 6E) are with predicted carboxyl-truncated transcripts (Fig. 6F). The failure to observe a macrophage migration defect despite such highly effective gene editing in macrophages indicates that either macrophages are more resilient to *trim33* gene-dosage reduction, or alternatively, and more likely, that *trim33* is not cell autonomously required for macrophage migration, as was previously suggested by observations in the *moonshine* mutant (Demy et al., 2017).

We also sought to assess the macrophage/neutrophil specificity of gene editing using functional and sequencing-based approaches, exploiting the fact that the *Tg(mpeg-Cas9)* line carried the *Tg(mpx:EGFP)* reporter marking neutrophils. First, we took advantage of the sensitivity of neutrophil migration to *trim33* knockdown to functionally assess whether neutrophils showed a migratory defect when macrophage gene editing was intended, knowing that 43.6% knockdown in neutrophils results in a migratory defect. Such a functional approach to assessing lineage specificity of gene editing has previously been employed for another neutrophil-specific gene-editing system (Wang et al., 2021). EGFP-positive neutrophils marked by the *Tg(mpx-EGFP)* transgene carried along with the *Tg(mpeg1-Cas9)* gene-editing system did not show a migratory defect when gRNA was delivered, despite the gRNA being proven to be potent by a concurrent positive control (Fig. 6G). Second, we looked for gene editing in FACS-purified EGFP-positive neutrophils from gRNA-injected *Tg(mpeg1-Cas)* embryos by Sanger and NGS sequencing. No gene editing was evident upon Sanger sequencing (Fig. 6C). Unexpectedly, however, NGS on a narrowly gated FACS-purified EGFP-expressing cell population (see Fig. S5G) detected carboxyl-truncating gene deletions in 48.4% of sequences (Dataset 2), in the same fish in which mCherry-positive macrophages showed 77.65% carboxyl-truncating gene edits. In the absence of a functional defect in neutrophils and clean Sanger sequencing chromatograms, we attribute this unexpected NGS result to a combination of technical issues: (1) a possible contribution from the previously reported low level of *mpeg1* promoter activity in *Tg(mpx:EGFP)*-positive neutrophils [Rougeot et al. (2019) reported a ratio of *mpeg1*:*mpx* transcripts in 5 dpf embryos of 0.3%]; (2) the likelihood that the gating strategy for neutrophils captured some cells of a low EGFP-expressing macrophage population, as described in the *Tg(-8mpx:EGFP)^uwm1^ line* (Fig. S5G) (Mathias et al., 2009); and (3) the possibility of preferential PCR amplification of deletion alleles across the 70 cycles of the two PCR reactions in the path to NGS sequencing. We excluded bar-coding misreads in the NGS as a cause of sample cross-contamination by re-running the neutrophil sample in a separate run to the macrophage sample. These points are considered further in the Discussion below.

Also of practical significance is that a higher proportion of *trim33* gene editing was achieved within *Tg(mpeg1-Cas9)* macrophages, despite the significantly lower level of Cas9 expression in this line than in the *Tg(mpx-Cas9)* line (Fig. 1G). This demonstrates unequivocally that the lower level of Cas9 expression in the *Tg(mpeg1-Cas9)* line is not a barrier to achieving macrophage lineage gene editing in the presence of an effective gRNA.

## DISCUSSION

Collectively, these results demonstrate functionally consequential, molecularly verified, gene editing in either the neutrophils or macrophages of zebrafish embryos, based on stable Cas9 expression restricted to each individual phagocyte lineage and transient gRNA delivery by microinjection.

In this system, *mpx* (Renshaw et al., 2006) and *mpeg1* (also known as *mpeg1.1*) (Ellett et al., 2011) promoters cell autonomously drive zebrafish phagocytic lineage-specific Cas9 transgene expression as a standalone, decoupled from gRNA delivery. This decoupling enables more flexibility of gene editing compared to a previously reported *lyzC*-driven neutrophil gene-editing approach (Zhou et al., 2018). Recently, Wang et al. (2021) described a refinement of this system, still employing plasmid-based transgene delivery for directing gRNA synthesis. In our system, gene editing in zebrafish embryos and early larvae can be achieved with relative ease by simple microinjection of synthetic gRNAs into the Cas9-expressing embryos without the need for subcloning. Furthermore, this approach permits easier gRNA multiplexing directed at multiple target sites, either within the same gene or at multiple loci.

That effective knockdown of genes would occur following synthetic gRNA microinjection was not a certain expectation, because RNA is a highly unstable molecule that is susceptible to degradation by ubiquitous RNases. Indeed, when gRNAs and Cas9 are co-injected for gene editing, they are customarily first complexed *ex vivo*, in part to enhance gRNA stability (Cong et al., 2013; Gagnon et al., 2014; Hwang et al., 2013; Jinek et al., 2012). Potentially contributing to the effectiveness of this system, the synthetic commercially procured gRNAs used here incorporated proprietary chemical modifications protecting from endogenous RNases. We have not tested whether delivery of *in vitro*-transcribed, non-modified gRNAs would be as effective. Another advantage of this approach is that it guarantees that the gRNA is present the moment the transgene promoter initiates Cas9 expression. There was a theoretical possibility that the *mpx* and *mpeg1* promoters would become active too late in development to engage enough residual microinjected gRNA molecules to achieve functionally significant gene editing. However, our functional data show that, with active, efficient, microinjected synthetic gRNAs, functionally consequential gene knockdown is achievable.

We also repurposed a *tol2*-flanked plasmid-based tRNA-based gRNA delivery system, in which single or multiple gRNAs are expressed constantly under the U6 promoter and are retained within the nucleoplasm. We hypothesized that this may have the advantage of providing for persistent, ongoing gene editing. However, only low-level gene editing was achieved. There are several possible explanations, including the following: (1) relatively inefficient expression from episomal or mosaic *tol2*-mediated genomic integrants in the microinjected F0 embryos; and (2) lower-level gRNA abundance or accessibility at the time of onset of Cas9 synthesis than that achieved by synthetic gRNA microinjection. Optimizations taking these factors into account could be employed for longer, more persistent gene editing throughout life, or in stable compound transgenic configurations.

We provide multiple examples showing that on-target gene editing across a PAM site can be recognized with Sanger sequencing, and that when it is highly efficient, an estimate of efficiency can be made that is comparable to that determined by NGS. This is consistent with observations from a previous study (Zhou et al., 2018). This is important practically because of the cost-saving implication that Sanger sequencing is good enough to determine whether a sufficient amount of on-target gene editing is occurring. However, as expected, Sanger chromatograms were unable to detect low levels of gene editing when present in samples (Fig. S6). NGS is required to detect low-level gene editing, and to determine the fraction of allelotypes predicted to cause functional gene disruption.

The other neutrophil gene-editing systems in zebrafish that have claimed neutrophil specificity have relied on the neutrophil lineage specificity of the *lyzC* promoter (Wang et al., 2021; Zhou et al., 2018). However, *lyzC* is also expressed in macrophages, although at lower levels. RNA sequencing showed that *lyzC* expression in FACS-sorted *mpeg1*-positive macrophages is present at 12% of the level in neutrophils (Rougeot et al., 2019). Additionally, Wang et al. (2021) examined macrophages in their neutrophil gene-editing line for a functional defect when only neutrophil *rac2* gene editing was expected and found no macrophage migratory defect. Wang et al. (2021) also performed deep sequencing on neutrophils and non-neutrophils from their system, finding 35.73% and 17.16% gene editing at two target sites in the GFP^+^ neutrophil population, and reporting that “in the GFP^−^ non-neutrophil population, a similar level of gene editing was not detected”.

We attempted to experimentally evaluate whether our new approach has the intended leukocyte-lineage specificity in embryonic/larval zebrafish by sequencing as well as functional approaches. The precision of sequencing approaches is limited by the specificity of the cell-purification strategies available. For the *Tg(mpx-Cas9)* system in which neutrophils express mCherry, the targeted locus was sequenced in FACS-purified mCherry-positive neutrophils and compared with all other residual cells in the embryos (Fig. 5C; gating strategy in Fig. S5F, NGS data in Dataset 1). The proportion of gene editing in neutrophils versus other cells was 1.45% versus 0% for *lbr* and 43.6% versus 15.75% for *trim33*. For the *Tg(mpeg1-Cas9)* system, both macrophages and neutrophils could be FACS purified, based on expression of different fluorophores from independent transgenes (Fig. 6C). The proportion of carboxyl-truncating gene editing in mCherry-positive macrophages versus EGFP-positive neutrophils was 77.65% versus 48.4% for *trim33*. This result is very surprising, given that this degree of gene editing would be expected to be evident in the corresponding Sanger sequencing chromatogram but was not, and the experimental evidence that there was no functional defect in neutrophil migration in *Tg(mpeg1-Cas9)* embryos, whereas 43.6% carboxyl-truncating *trim33* mutations resulted in a reproducible migration defect in the neutrophils of *Tg(mpx-Cas9)* embryos.

We therefore hypothesize that these NGS sequencing results at least in part reflect technical issues in preparing samples for NGS analysis. At the low cell numbers involved, a few FACS mis-sorted cells could have a major effect on the pool of template DNA for NGS (FACS gating strategies are detailed in Fig. S5). Another potential factor is preferential amplification of deletion alleles during PCR amplification in the NGS workflow. Mathematically, at the level of gene editing observed in the intended lineage, only a 1:100 mis-sort of cells and 7% difference preferentially favouring amplification of a deletion allele across 35 cycles of PCR would result in distortion of the relative levels of minor transcript representation to the levels we report. In fact, there are two PCR reactions amounting to 70 PCR cycles separating the purified cells from the NGS result.

To date, it has not been customary in lineage-specific gene-editing studies in zebrafish to experimentally assess editing in other lineages by NGS, both for gene editing in neutrophils (Zhou et al., 2018) and for gene editing in other tissues (Ablain et al., 2015; Di Donato et al., 2016; Dong et al., 2009; Savage et al., 2019; Yin et al., 2015). Our experience suggests that NGS can be useful for confirming the nature of gene editing that is occurring, but to be a test of lineage/tissue specificity it requires infallible cell purification methods. This limitation of FACS purification of cell populations for sequencing in this setting has been recognized by others previously (Ablain et al., 2015; Di Donato et al., 2016).

Despite these caveats, we present our two systems as the most rigorously characterized ‘leukocyte lineage-specific’ systems that have been developed in zebrafish to date. We strongly recommend that, for both systems, investigators include appropriate functional and sequencing specificity controls in their experiments rather than make presumptions. Sequence corruption starting around the PAM site in Sanger chromatograms prepared from leukocyte DNA can demonstrate that on-target gene editing has occurred in the intended phagocyte lineage, but is too insensitive to exclude gene editing in the other phagocyte type. NGS is useful for defining the nature and diversity of heterogeneous gene edits that occur. However, to be used to exclude off-target gene editing, NGS requires infallible cell-purification methods. In the particular case of phagocyte gene editing, the availability of independent systems designed for preferential gene knockdown in either neutrophils or macrophages enables any concern about leakiness of gene knockdown in the intended lineage of either line to be functionally directly evaluated by experimental knockdown in the other, a functional specificity control that we have demonstrated here for *trim33* knockdown (Fig. 6G). We have used the approach of employing both systems together in our recently published application of these tools, showing the functional consequences of *Tg(mpeg1-cas9)* NAMPT knockdown in macrophages, controlled by no effect from *Tg(mpx-Cas9)* NAMPT knockdown in neutrophils (Ratnayake et al., 2021). Ultimately, the accumulated experience of multiple investigators using these tools will best resolve uncertainty about their specificity and utility.

We targeted *trim33* to demonstrate the functional utility of these systems in defining the cell autonomy of a leukocyte phenotype*.* The *trim33* mutant *moonshine* was previously reported to show defects in both neutrophil and macrophage migration to an injury site (Demy et al., 2017). In this study, two independent lines of evidence ascribe this defect to *trim33* (mutant and morphant studies, although rescue experiments were not reported), so we selected *trim33* knockdown as a test of utility for achieving a functional outcome from our lineage-targeted system. Our lineage-targeted *trim33* knockdown impaired neutrophil recruitment to the site of the caudal fin wound, replicating the neutrophil-specific cell-autonomous requirement of *trim33* for neutrophil migration, which in the previous report was also demonstrated to be cell autonomous in transplantation experiments (Demy et al., 2017). In contrast, in our system, highly efficient, molecularly verified, macrophage-lineage *trim33* knockdown did not alter migrating macrophages numbers at the wound, indicating that this defect is unlikely to be cell autonomous. This is consistent with the previous report, which did not explicitly evaluate the macrophage migration defect by transplantation experiments. Furthermore, by assessing neutrophil migration in *Tg(mpeg1-Cas9)* knockdown embryos, we have shown that there is no *trim33*-requiring macrophage-dependent secondary effect on neutrophil migration (Fig. 6G). In light of these findings, we hypothesize that the macrophage migration defect in *moonshine* mutants is secondary to the defect in neutrophil migration, as neutrophils are the earlier-arriving cell type. Verifying this will require further experiments. Although this is beyond the scope of our studies, it does highlight the need for experimental rigor in ascribing phenotypes to cell-autonomous gene activity and the limitations of germline mutants in this regard.

Transplantation remains the gold standard for assessing cell autonomy, but, in zebrafish systems, the available developmental, homochronic and heterochronic options are technically challenging and carry caveats. With this new system, we provide a genetically flexible and comparatively easier resource for testing cell-autonomous gene function in either neutrophils and macrophages in zebrafish embryos. It will be a valuable addition to the experimental toolbox available to zebrafish leukocyte biologists.

## MATERIALS AND METHODS

### Animal ethics, husbandry and alleles

Zebrafish (*Danio rerio*) experiments were conducted under protocols approved by the Monash University Animal Ethics Committees (MARP-2015-094/14375 and 17270), and in accordance with the National Health and Medical Research Council (NHMRC) guidelines (NHMRC, 2013). Zebrafish were housed, raised and managed at the Monash University AquaCore Research Facility. Embryos were kept in Methylene Blue-treated E3 medium (5 mM NaCl, 0.17 mM KCl, 0.33 mM CaCl_2_, 0.33 mM MgSO_4_, equilibrated to pH 7.0) and transferred (12 hours post-fertilization) to egg water [0.06 g/l salt (Red Sea, Sydney, Australia) containing 0.003% 1-phenyl-2-thiourea (Sigma-Aldrich)]. Zebrafish lines used were various compound transgenic lines carrying the following alleles: WT Tübingen (TU) (Max-Planck-Institut für Entwicklungsbiologie, Tübingen, Germany), *Tg(mpx:Kal4TA4)^gl28^* (Okuda et al., 2015), which carries a *cryaa:EGFP* backbone marker conferring green eyes, *Tg(mpeg1.1:Gal4FF)^gl25^* (Ellett et al., 2011), *Tg(mpx:EGFP)^i114^* (Renshaw et al., 2006), *Tg(UAS-E1b:Eco:NfsB-mCherry)^c26^*^4^ (Zebrafish International Stock Center, Eugene, OR, USA) (Davison et al., 2007), and the new *Tg(4xUAS:NLS-Cas9, cmlc2:RFP)^gl37^* and *Tg(4xUAS:NLS-Cas9, cryaa:EGFP)^gl36^* lines.

### *UAS:Cas9* and gRNA plasmids

#### 4xUAS:NLS-Cas9, cmlc2:RFP

*UAS:Cas9* plasmids were constructed by MultiSite (three-element) Gateway cloning (Kwan et al., 2007). Cas9 was amplified from pT3TS-nCas9n (Addgene plasmid #46757) with primers nCas9n-attB1Fwd: 5′-GGGGACAAGTTTGTACAAAAAAGCAGGCTGCCACCATGGCTTCTCCACCTAAGAAGAA-3′/nCas9n-attB2RRev: 5′-GGGGACCACTTTGTACAAGAAAGCTGGGTAGTGGTAACCAGATCCGCGGT-3′ using PCR, and inserted into the donor vector pDONR™221 to generate a middle-entry vector pME-nCas9n by BP cloning reaction. *cmlc2*-TagRFP was amplified from pDestTol2CG3 (primers CG3-Hind3-Fwd: 5′-GATCTAAAGCTTAAATCAGTTGTG-3′/TagRFPFwdrc-cmlc2Rev1: 5′-CCTTAGACACCATGGTGGCGGGTCACTGTCTGCTTTGCTGTT-3′) and pTagRFP-N (primers cmlc2RevrcTagRFPFwd: 5′-AACAGCAAAGCAGACAGTGACCCGCCACCATGGTGTCTAAGG-3′/CG3-KpnIRev: 5′-GGGGTACCTAAGATACATTGATGAGTTTGGAC-3′) by an overlap extension PCR, which replaced the *cmlc2* GFP in the donor vector pDestTol2CG3 and generated the new donor vector pDestTol2CR3. pDestTol2CTagRFP-4xnrUAS-nCas9n (*4xUAS:NLS-Cas9, cmlc2:RFP*, plasmid map in Fig. S7A) was constructed with entry vectors p5E-4xnrUAS, pME-nCas9n and p3E-polyA and destination vector pDestTol2CR3 by LR cloning reaction.

#### 4xUAS:NLS-Cas9, cryaa:EGFP

pDestTol2pACryGFP-4xnrUAS-nCas9n (*4xUAS:NLS-Cas9,cryaa:EGFP*, plasmid map in Fig. S7B) was constructed with entry vectors p5E-4xnrUAS, pME-nCas9n and p3E-polyA and destination vector pDestTol2pACryGFP (Addgene plasmid #64022, deposited by Joachim Berger) by LR cloning reaction. All plasmid sequences are available upon request.

#### *lbr* gRNA

To construct the plasmid-based gRNA delivery system (Fig. S7C), ubb:Cas9 was removed from TSKK27-pT2TS-ubb:Cas9;u6c:Dr-tRNAGly(GCC) single-guide RNA (sgRNA) scaffold (Shiraki and Kawakami, 2018) by PCR using primers U6cFwd: 5′-TGGGGGATATTATGAAGGGCC-3′/U6cRev: 5′-AATACTCAAGTACAATTTTAATGG-3′.

Template plasmid DNA in the PCR reaction was digested using Dpn1 at room temperature overnight. Dpn1 was deactivated at 80°C for 20 min. The 25 µl digested PCR product was phosphorylated using T4 kinase, at 37°C for 30 min in the following reaction mix: 0.5 µl kinase buffer, 1 µl linearized plasmid DNA, 0.5 µl T4 kinase and 3 µl deionized water. Blunt-end PCR fragment was circularised using T4 ligase at room temperature for 60 min by adding the following to the T4 kinase reaction mix: 3.5 µl deionized water, 1 µl T4 ligase buffer and 0.5 µl T4 ligase. The entire mix was transformed in 100 µl DH5-alpha competent *Escherichia coli*, and the plasmid purified using QIAprep Spin Miniprep kit (QIAGEN) and confirmed by Sanger sequencing. *lbr* plasmid gRNA was generated as described previously (Shiraki and Kawakami, 2018) by PCR with primers Gly_lbrpgRNA1Fwd: 5′-GATTCCCGGCCAATGCAGAACGGGAGCGACTGCGACGGTTTAAGAGCTATGCTGGAA-3′ and Thr_lbrpgRNA2Rev: 5′-CAGCATAGCTCTTAAACCGTCGCAGTCGCTCCCGTTCAGGCACCGCTGGGATTCGAAC-3′ and template vector DR-tRNA^Thr^(AGT). Amplified *lbr* gRNA was then inserted into u6c:Dr-tRNAGly(GCC) sgRNA scaffold using In-Fusion cloning technique and confirmed by Sanger sequencing.

### Transgenesis and breeding

*Tg(4xUAS:NLS-Cas9,cmlc2:RFP)* and *Tg(4xUAS:NLS-Cas9,cryaa:EGFP)* plasmids were co-injected with transposase mRNA (15 ng/μl) into one-cell-stage embryos of either *Tg(mpx:Kal4TA4;UAS:NfsB-mCherry)* or *Tg(mpeg1:Gal4FF; UAS:NfsB-mCherry;mpx:EGFP)* compound transgenic lines. To select F0 founders and propagate stable transgenic lines, colonies were managed by selection at each generation for co-segregation of mCherry leukocytes (indicating the presence of both a *Gal4* driver and the *UAS:NfsB-mCherry* effector) and the respective Cas9 transgene backbone marker, either red heart (*cmlc2:RFP*) for mCherry-labelled neutrophils or green eyes (*cryaa:EGFP*) for mCherry-labelled macrophages. In addition, for the line with *4xUAS:NLS-Cas9* targeted to macrophages, the breeding strategy maintained selection to retain the *Tg(mpx:GFP)* neutrophil reporter transgene. The outcome was two compound transgenic lines with expression of Cas9 in either neutrophils or macrophages, as follows: (1) *Tg(mpx:Kal4TA4;UAS:NfsB-mCherry;Tg(4xUAS:NLS-Cas9,cmlc2:RFP)*, with mCherry- and Cas9-expressing neutrophils and a red heart (and also green eyes from the *mpx:KalTA4* transgene backbone tracer); and (2) *Tg(mpeg1:Gal4FF;UAS:NfsB-mCherry;4xUAS:NLS-Cas9,cryaa:EGFP;mpx:EGFP)*, with mCherry- and Cas9-expressing macrophages, EGFP-expressing neutrophils without Cas9, and green eyes. For simplicity, these two compound transgenic lines are referred to as *Tg(mpx-Cas9)* and *Tg(mpeg1-Cas9)*, respectively. Table 1 presents full details of the compound transgenic lines used in these studies and backbone markers used to facilitate their genetic selection.

### PCR genotyping and phenotyping

For genotyping PCRs, HotShot method (Meeker et al., 2007) was used to extract genomic DNA from zebrafish embryos. For whole-embryo Cas9 expression assessment by RT-PCR, ∼40 embryos per group were processed for RNA extraction using TRIZol reagent (Thermo Fisher Scientific).

### Leukocyte collection and purification for PCR

For leukocyte Cas9 expression assessment by RT-PCR, ∼100 pooled embryos or three adult WKMs were homogenized in FACS medium (0.9× PBS+5% heat-inactivated foetal bovine serum) using a 21G or 23G needle (Terumo^®^). Leukocytes were FACS purified and processed for RNA extraction using TRIZol reagent (Thermo Fisher Scientific).

### RT-PCR

cDNA templates for RT-PCR were synthesized using a SuperScript™ IV First-Strand Synthesis System (Thermo Fisher Scientific). PCR used primers (Table S1) and Phusion High-Fidelity DNA Polymerase (Thermo Fisher Scientific), run in a T100 thermal cycler (Bio-Rad) as follows: initial denaturation (95°C, 30 s), 30 cycles of denaturation (95°C, 30 s), annealing (66°C, 30 s) and initial extension (72°C, 30 m s); final extension (72°C, 5 min). Amplified PCR products were resolved by electrophoresis in ethidium bromide-stained 1.5% agarose gels.

### RT-qPCR

For Cas9 expression assessment by RT-qPCR, 42-50 pooled embryos at 3, 4 and 5 dpf were processed for RNA extraction using TRIZol reagent (Thermo Fisher Scientific). A SuperScript™ VILO™ cDNA Synthesis Kit (Thermo Fisher Scientific) was used according to the manufacturer's guide to synthesize cDNA templates for the RT-qPCR. cDNA samples were diluted 1:20 and added to an equal volume of a LightCycler^®^ 480 SYBR Green I Master (Roche). RT-qPCR was done in a 10 µl reaction volume using LightCycler^®^ 480 II (Roche) with *ppial* (also known as *ppiab*) as housekeeping gene (van der Vaart et al., 2013; van Soest et al., 2011). Cas9 transcript was not detected from 17/18 of total no template and WT template samples. Relative Cas9 expression in 3-5 dpf *Tg(mpx-Cas9)* and *Tg(mpeg1-Cas9)* embryos was calculated by comparative CT (2^-ΔCT^) method, normalizing data with the housekeeping gene *ppial* and comparing transgenic Cas9 embryos with WTs (Schmittgen and Livak, 2008).

### CRISPR-Cas9 gene editing

Tests for CRISPR-Cas9 gene editing used either previously characterized gRNAs (Manley, 2019) or new gRNAs designed using Integrated DNA Technologies (IDT) custom Alt-R CRISPR-Cas9 guide and MENTHU (Ata et al., 2018) gene-editing software (Table S2). All gRNAs commercially synthesized (IDT™), included both crispr RNA (crRNA) and trans-activating crispr RNA (tracrRNA) sequences, and incorporated proprietary chemical modifications to enhance their stability. crRNA (10 nmol) and tracrRNA (20 nmol) were mixed at 1:2 (v/v), denatured at 95°C for 5 min and allowed to anneal at room temperature for 15-30 min. gRNA (annealed crRNA and tracrRNA) were microinjected directly or as a mixture of 1 µl synthetic gRNA (or 12.5 ng for plasmid gRNA), 0.5 µl 100 µg Alt-R^®^ S.p. Cas9 Nuclease V3 (IDT™) and 8.5 µl duplex buffer (IDT™).

Newly designed commercially synthesized and tRNA-based plasmid gRNAs were first co-injected with Cas9 enzyme into one-cell-stage WT embryos and validated for on-target activity by T7 endonuclease (Alt-R Genome Editing Detection Kit, IDT™) and/or Sanger sequencing analysis across the target sequence site in 3 dpf embryos. gRNAs with validated on-target activity were deemed suitable for injection into one-cell-stage *Tg(mpx-Cas9)* and *Tg(mpeg1-Cas9)* embryos. In some experiments, 3-5 dpf neutrophils and/or macrophages from gRNA-injected *Tg(mpx-Cas9)* and *Tg(mpeg1-Cas9)*, respectively, embryos were purified by FACS for genomic DNA analysis.

### FACS of neutrophils and macrophages for sequencing

To purify leukocytes from embryos, embryos were anaesthetized using tricaine methanesulfonate (Sigma-Aldrich), and a fine surgical blade (Swann-Morton^®^) was used to divide the embryo into two parts, cutting caudal to the heart. This permitted the heart and eyes to be discarded, along with reporter genes for which expression could confound the purity of FACS-purified leukocytes. Pooled embryos were prepared as a single-cell suspension using a combined enzymatic and mechanical dissociation protocol (Bresciani et al., 2018). Neutrophils and macrophages were isolated using Monash University FlowCore's Arial Fusion or Influx 1 cell sorter (BD Biosciences), gating by mCherry. In some experiments, non-mCherry-positive cells from *Tg(mpx-Cas9)* embryos, or EGFP-positive cells from *Tg(mpeg1-Cas9)* embryos, were also sorted as controls. Gating strategies for all collections are displayed in Fig. S5. Cells were sorted directly into 8 mM NaOH for DNA extraction by the HotShot method.

### Sanger DNA sequencing

PCR-amplified DNA fragments for sequencing (Table S1) were purified using QIAquick^®^ Gel Extraction or PCR purification kit (QIAGEN), according to the manufacturer's guide for PCR product purification and sequencing. Sanger sequencing was done at Monash University Micromon; NGS was done at Peter MacCallum Cancer Centre, Melbourne.

### NGS

PCR amplification and NGS of exon 2 (Transcript ID: ENSDARE00000118961) of *lbr* and exon 8 of *trim33* (Transcript ID: ENSDART00000020116.9) were performed in duplicate using single amplicons amplified with locus-specific primers containing the Fluidigm (South San Francisco, CA, USA) universal forward and reverse sequencing tags (CS1 and CS2) as indicated in Table S1 and using methodology previously published (Blombery et al., 2018). The PCR amplification was performed on ∼500 ng of genomic DNA using a FastStart High-Fidelity PCR System (Roche). The PCR conditions consisted of an initial denaturation step of 95°C for 7 min, followed by 35 cycles of 95°C for 30 s, 63°C for 30 s and 72°C for 30 s, and a final elongation step at 72°C for 7 min. The harvested products from this first PCR reaction amplifying the region of interest were diluted 1:100 with water and spiked in alongside routine diagnostic targeted amplicon panels (48 samples total), which were also diluted 1:100. This 1:100 dilution for all samples was added to a mastermix containing 48 different barcodes (Fludigm) for indexing PCR prior to sample pooling and sequencing. Uniquely indexed samples were pooled, and the resulting library was purified using an Agencourt AMPure XP system (Beckman Coulter, Brea, CA, USA). The resultant library was quantified on a TapeStation 2200 (Agilent Technologies, Santa Clara, CA, USA). Libraries were denatured and diluted, as per the manufacturer's instructions, and 150- base pair (bp), paired-end sequencing was performed on an Illumina (San Diego, CA, USA) MiSeq sequencer using MiSeq version 2 chemistry.

### Bioinformatics and variant calling

The genome reference build GRCz11 for zebrafish was downloaded from Ensembl (http://www.ensembl.org/). Raw reads were de-multiplexed and processed using an in-house bioinformatics analysis pipeline. Bcl2fastq (v2.20.0.422) was used to perform sample de-multiplexing and to convert base call (bcl) files generated from the MiSeq instrument into FASTQ files containing short-read data. FASTQ data were trimmed for CS tags in 3′ end using cutadapt tool (v1.9.1). Trimmed data were then aligned to the whole-genome reference using bwa-mem aligner (bwa 0.7.17) to generate the binary alignment map (BAM) file (Li, 2013). The BAM file was then filtered for primary alignments as required by the downstream variant caller. The Pisces Illumina Variant caller (5.2.10.49) in somatic mode was used to call variants with a variant allele frequency (VAF) >1% within the two amplicon regions of interest (*trim33*, 18:44832509-44832770; *lbr*, 20:36407311-36407554). Variants detected in the WT sample were excluded from further analysis. Sequence reads for the remaining variants were manually inspected to exclude technical artefact and assess variant phase. The average VAF of the sample duplicates was calculated. One *lbr* variant (20:g.36407345G>A) (Dataset 1) and two *trim33* variants (chr8:g.11004484C>A, chr8:g.11004454T>A) (Dataset 2) were dismissed as strain-based polymorphisms. The incidence of these polymorphisms varies between the samples because of different parental pedigrees of each pool.

### Phagocyte chemotaxis assay

Using a size-22 sterile surgical blade (Swann-Morton^®^), the tip of the caudal fin distal to the end of notochord was excised from phenotypically normal anaesthetized 3 dpf embryos. Injured embryos were kept for 3.5 h for neutrophil and macrophage recruitment to the wound site, before fluorescent imaging using an MVX10 microscope (Olympus). Photomicrographs used for scoring the 647 embryos of Figs 3, 5 and 6 and Fig. S3 are available on request, verifying their normal gross morphology.

### Microscopy and image handling

Microscopic images were captured with an Olympus DP72 camera and CellSens software version 1.11. Images were edited to improve contrast using Adobe Photoshop and adjusted to size using Adobe Illustrator. Phagocyte number at the wound was manually counted for each embryo and tabulated for analysis.

### Statistical considerations and analysis

For quantitative analyses, group sizes of >10 animals/experiment were intended but determined by the number of embryos laid on the day, with two to three replicates. Anticipated effect sizes were not pre-specified. The contributions of individual experiments to pooled data are colour coded in the figures. Embryos were randomly assigned to experimental groups. Scoring was not blinded. Descriptive and analytical statistics were done using Prism 8 Version 8.3.1(332) (GraphPad Software, CA, USA). Data are mean±s.d. with *P*-values generated from two-tailed unpaired Student's *t*-tests for continuous variables and chi-square tests for categorical variables. *P*-values <0.05 were considered significant. Data points ≥4 s.d. from the mean were treated as outliers (applied only to two of 141 values in Fig. 6G).

## Supplementary Material

Supplementary information
