# Supplementary material for: Transient, flexible gene editing in zebrafish neutrophils and macrophages for determination of cell-autonomous functions
Source: Dis Model Mech. 2021 Jul 23;14(7):dmm047431. doi: 10.1242/dmm.047431 (PMC8319549; doi:10.1242/dmm.047431)
Supplement: Supplementary information [file dmm-14-047431-s1.pdf]

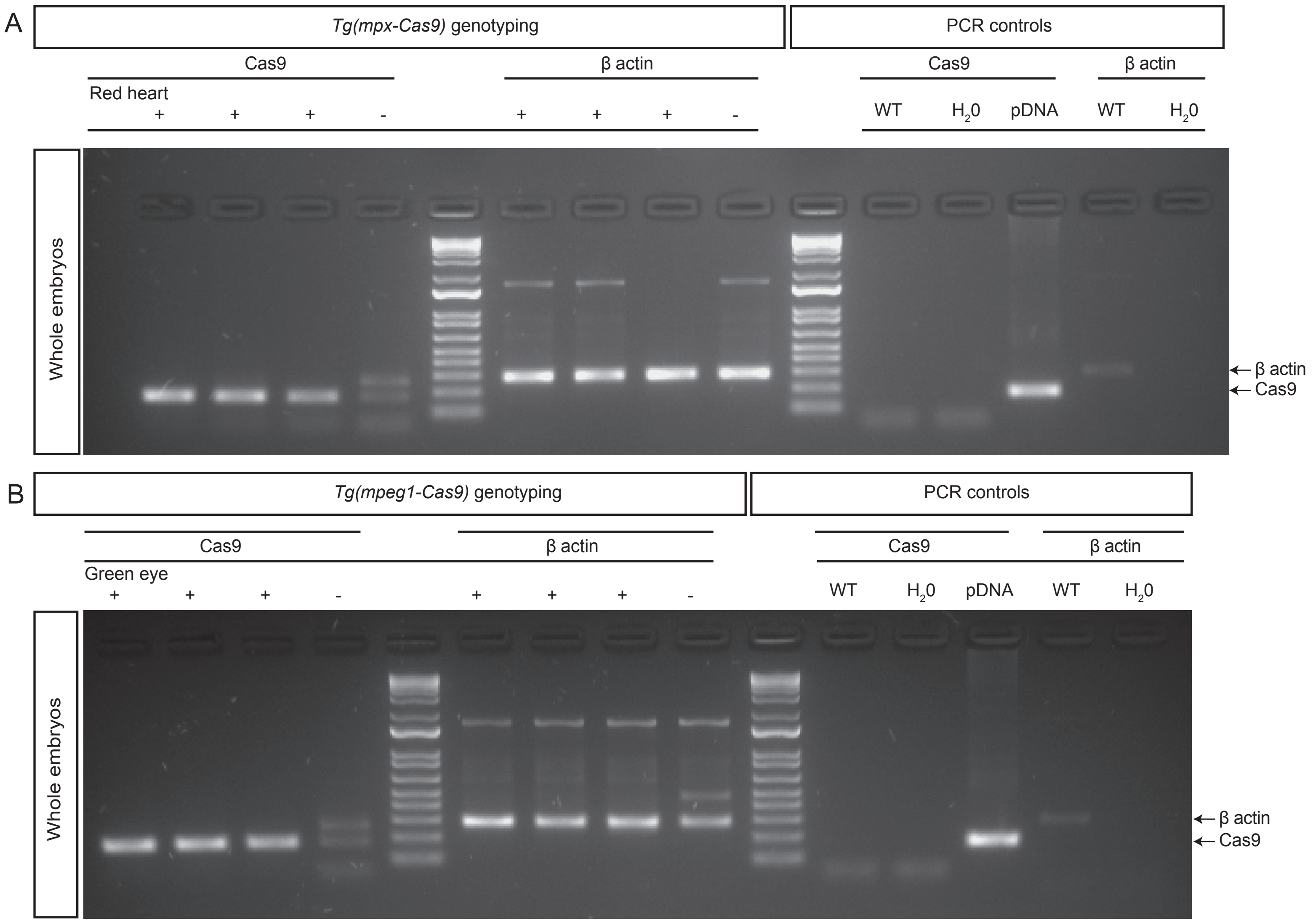

Figure S1. Correlation of Cas9 genotyping with presence of tracer transgene in *Tg(mpx-Cas9*) and *Tg(mpeg1-Cas9*) zebrafish lines

(A,B) Genotype confirmation of Cas9 cDNA segregation with Cas9 transgene plasmid backbone marker. Cas9-PCR genotyping of individual F1 *Tg(mpx-Cas9)* and F2 *Tg(mpeg1-Cas9)* zebrafish embryos selected for mCherry expression in phagocytes, and also presence/absence of the Cas9 transgene plasmid backbone marker (either red heart Tg(mpx-Cas9), or green eye *Tg(mpeg1-Cas9*).

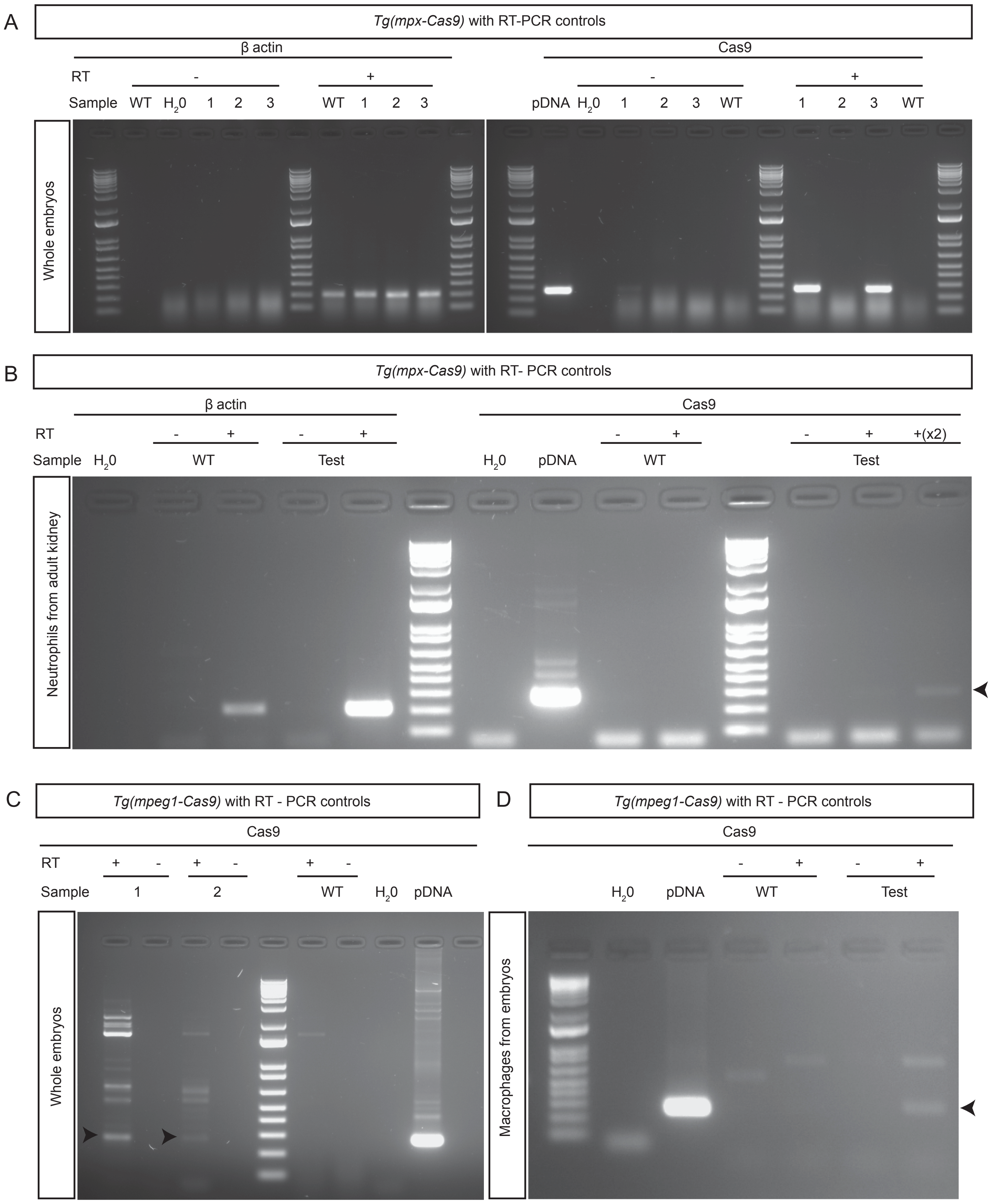
A
Tg(mpx-Cas9) with RT-PCR controls
β actin
Cas9
RT
-
+
-
+
Sample WT H20 1 2 3 WT 1 2 3
pDNA H20 1 2 3 WT 1 2 3 WT
Whole embryos
B
Tg(mpx-Cas9) with RT- PCR controls
β actin
Cas9
RT
- + - +
- + - + +(x2)
Sample H20 WT Test
H20 pDNA WT Test
Neutrophils from adult kidney
C
Tg(mpeg1-Cas9) with RT - PCR controls
Cas9
RT + - + - + -
Sample 1 2 WT H20 pDNA
Whole embryos
D
Tg(mpeg1-Cas9) with RT - PCR controls
Cas9
- + - +
H20 pDNA WT Test
Macrophages from embryos

Figure S2. Molecular confirmation of Cas9 mRNA expression in *Tg(mpx-Cas9*) and *Tg(mpeg1-Cas9*) zebrafish lines.

(A) Cas9-RT-PCR of F2 *Tg(mpx-Cas9)* whole embryos, confirming Cas9 expression in 2/3 test embryos. The negative result in sample #2 is interpreted contextually as either an embryo sorting or PCR failure rather than a true negative result.

(B) Cas9-RT-PCR of neutrophils FACS-purified from F1 *Tg(mpx-Cas9)* adult whole kidney marrow, showing detection of Cas9-cDNA in the sample with double-dose template (x2).

(C,D) Cas9-RT-PCR of *Tg(mpeg1-Cas9)* whole F2 embryos (C) and macrophages FACS-purified from F4 pools (F), showing detection of Cas9-cDNA in the samples. In this analysis, the housekeeping gene sample-quality control PCR failed and the samples were exhausted precluding further analysis, but the absence of this sample integrity control does not undermine the observation of specific Cas9 positivity in the test samples. Arrowheads in B,C,D indicate faint Cas9 PCR products. Throughout these analyses PCR controls included are: wild type template (WT); water template (H_20); template-containing plasmid DNA positive control (pDNA); ® actin PCR for sample integrity (® actin); for RT-PCRs, samples processed without and with reverse transcriptase (RT+ and RT-).

Disease Models & Mechanisms • Supplementary information

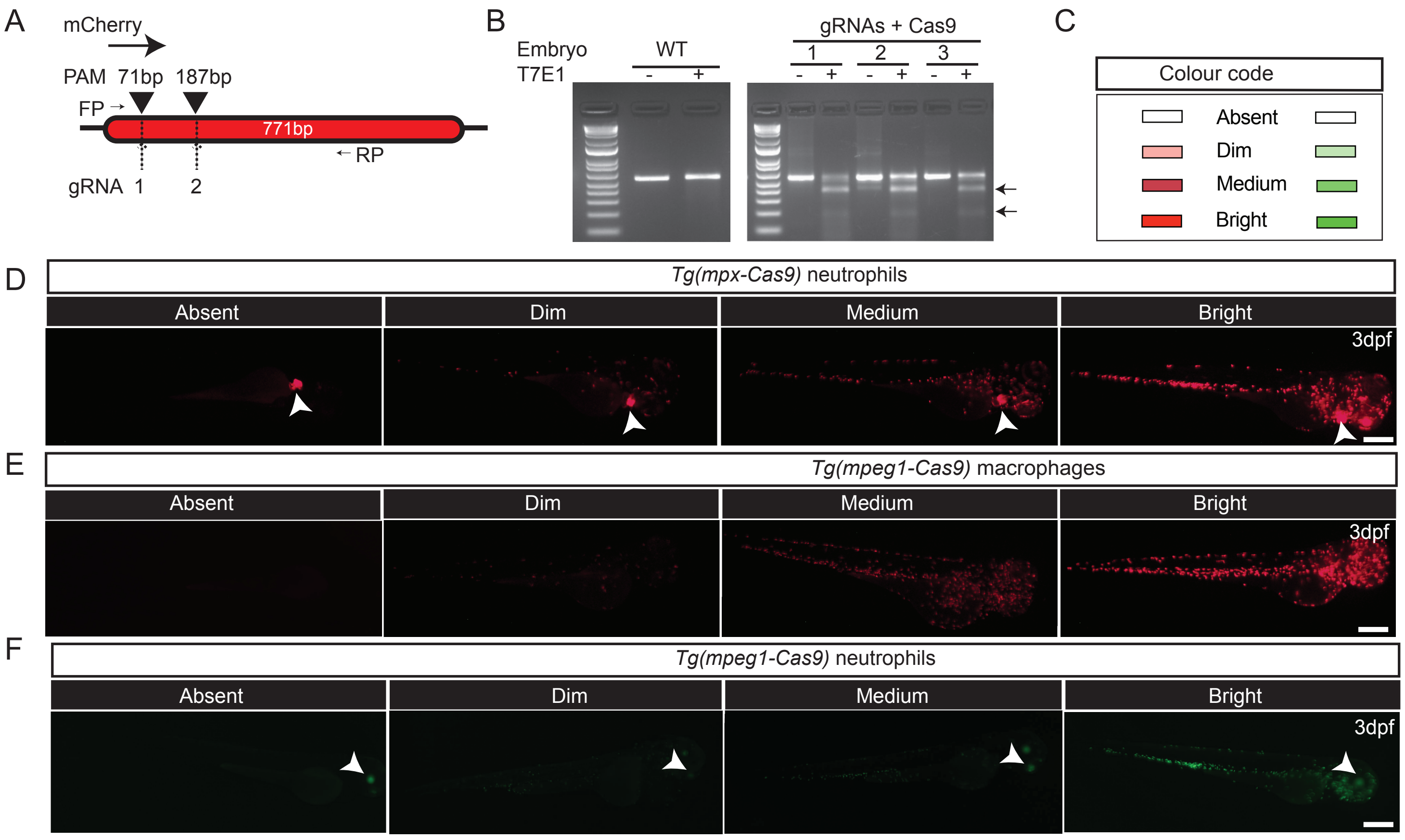

Figure S3. mCherry reporter gene knockdown in leukocyte lineage-specific Cas9-expressing zebrafish embryos.

(A) mCherry cDNA annotated to show the two targeted protospacer adjacent motif (PAM) gRNA sites.

(B) Confirmation of on-target activity of multiplexed mCherry-gRNA pairs demonstrated by T7 endonuclease digestion of heteroduplexes in mCherry PCR product (arrowed).

(C) Categorical scoring scale for mCherry and EGFP fluorescent leukocytes, as exemplified in (D-F), pertaining to Fig 1H-J.

(D-F) representative images of absent, dim, medium and bright leukocyte fluorescence categories for the reporter lines shown. White arrows indicate red heart (D) and green eye (F). For details of full genotypes, see Table 1.

Scale bar: 300 µm.

Disease Models & Mechanisms • Supplementary information

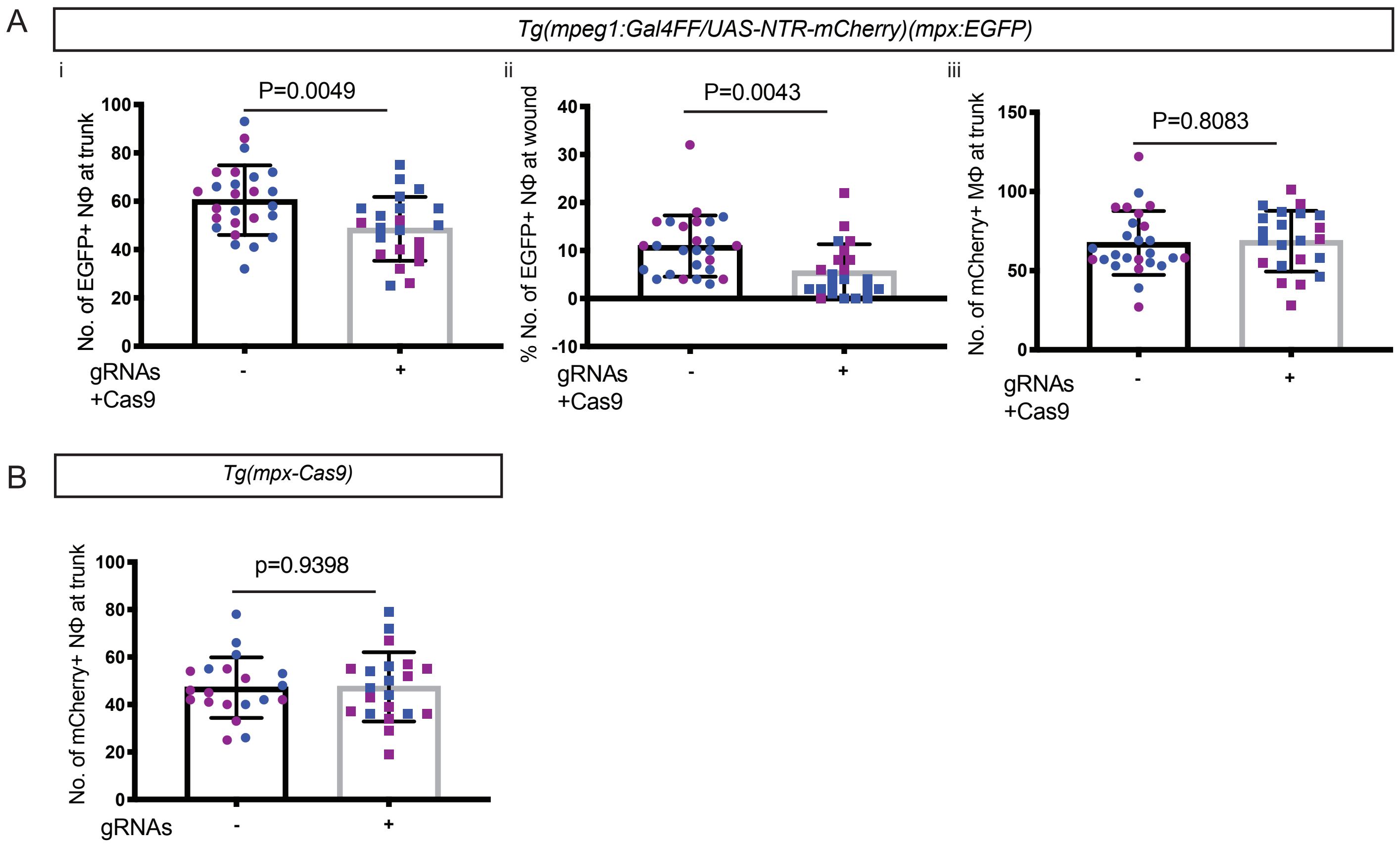

Figure S4. Basal number of leukocytes in trim33 crispant zebrafish embryos.

(A) Quantification of neutrophils (i, ii) and macrophages (iii) in the trunk of trim33 gRNA+Cas9 microinjected zebrafish embryos, compared to wild type, WT.

(B) Quantification of neutrophil number in the trunk of trim33 gRNA microinjected *Tg(mpx-Cas9)* zebrafish embryos, compared to WT.

Unpaired two-tailed Student T-test of pooled data from two independent experiments indicated by different colours.

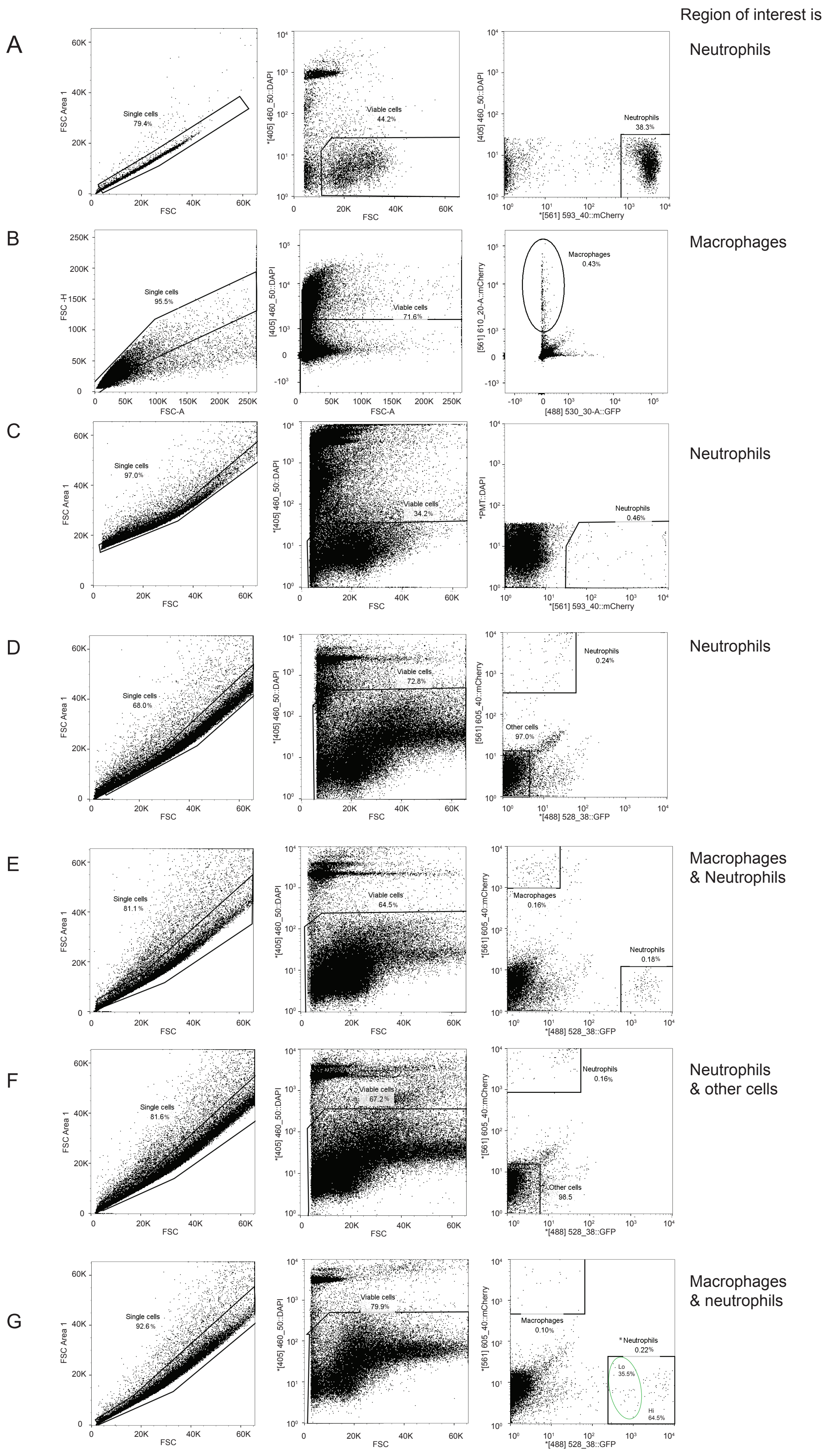

Disease Models & Mechanisms • Supplementary information

Figure S5. FACS gating strategies for purification of neutrophils, macrophages and control cell populations.

Gating strategy showing single, viable, mCherry (neutrophils or macrophages) and GFP (neutrophils) positive cells from single cell suspension

(A) 373,000 neutrophils purified from 3 pooled adult WKM, and

(B) 5, 000 macrophages purified from embryos (n= ~100) for RT-PCR and

(C-G) 743 neutrophils, 128 neutrophils, 1,170/1,231 macrophages/ neutrophils, 101/52,177 neutrophils/other cells and 31/72 macrophages/neutrophils purified from embryos (n= ~5-15) for sequencing.

A and B contribute to data generated in Fig S2B and S2D respectively; C, D, E, F, G contribute to data generated in Fig 2iii, 2iv, 4, 5 & 6 respectively. *This region contains both neutrophils and macrophages (Mathias et al., 2009): Lo indicates low GFP reporter transgene expressing cells (which have been demonstrated to be macrophages) and Hi indicates high GFP expressing cells (which are neutrophils).

A

Neutrophils from *Tg(mpeg1-Cas9)* line

```
Position                                    123456789123456
WT                    TCCTCCTCCTCCACCACCACCACCTCCGCCGCCGCCACCGTCCACCCCAGCGGACAGCACCGCAGCTGCCGC
MPEG_GFP c.284_311del TCCTCCTCCTCCACCACCACCACGGACAGCACCGCAGCTGCCGC(14.4%)
         c.276_300del TCCTCCTCCTCCACCTCCACCCCAGCGGACAGCACCGCAGCTGCCGC(11.1%)
         c.290_300del TCCTCCTCCTCCACCACCACCACCTCCGCTCCACCCCAGCGGACAGCACCGCAGCTGCCGC(8.3%)
         c.297_307del TCCTCCTCCTCCACCACCACCACCTCCGCCGCCGCCCAGCGGACAGCACCGCAGCTGCCGC(5.3%)
```

Nucleotide propotion (%) \ Position	1	2	3	4	5	6	7	8	9	1	2	3	4	5	6
A =	11.1	0	25.5	0	8.3	11.1	0	14.4	60.9	13.6	11.1	0	0	0	13.6
G =	14.4	0	5.3	11.1	0	19.7	0	0	25.5	0	0	25.5	13.6	13.6	25.5
T =	0	8.3	0	0	0	0	0	0	0	0	14.4	0	60.9	11.1	0
C =	74.5	91.7	66.2	88.9	91.7	80.6	100	85.6	13.6	86.4	74.5	86.4	25.5	73.5	60.9

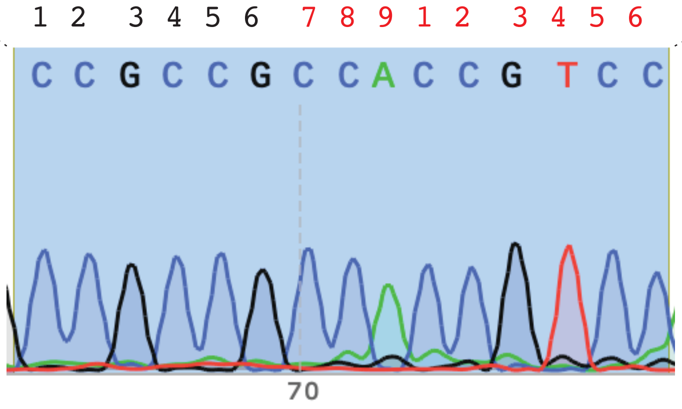

B

Other cells from *Tg(mpx-Cas9)* line

```
Position                                               123456789123456
WT                               TCCTCCTCCTCCACCACCACCACCTCCGCCGCCGCCACCGTCCACCCCAGCGGACAGCACCGCAGCTGCCGC
MPX_-VECHERRY c.284_311del TCCTCCTCCTCCACCACCACCACGGACAGCACCGCAGCTGCCGC(9.2%)
              c.273_300del TCCTCCTCCTCCTCCACCCCAGCGGACAGCACCGCAGCTGCCGC(5.7%)
```

Nucleotide propotion (%) \ Position	1	2	3	4	5	6	7	8	9	1	2	3	4	5	6
WT=	85.1	85.1	85.1	85.1	85.1	85.1	85.1	85.1	85.1	85.1	85.1	85.1	85.1	85.1	85.1
A =	0	0	14.9	0	0	0	0	14.9	0	0	0	0	0	0	0
G =	14.9	0	85.1	0	0	100	0	0	14.9	0	0	14.9	0	0	14.9
T =	0	0	0	0	0	0	0	0	0	0	14.9	0	85.1	0	0
C =	85.1	100	0	100	100	0	100	85.1	0	100	85.1	0	14.9	100	85.1

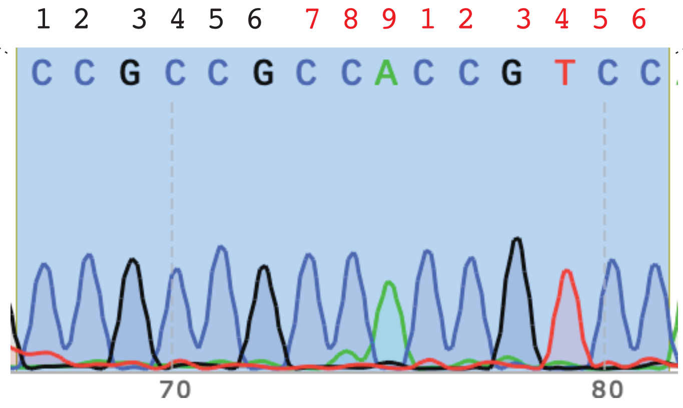

Disease Models & Mechanisms • Supplementary information

Figure S6. Two examples of predicted proportional representations of nucleotides in Sanger sequencing chromatograms, given variant incidences in next generation sequencing analyses.

(A) Analysis for mpx:EGFP-positive neutrophils in *Tg(mpeg1:Cas9)* line, injected with *trim33*-gRNA. The variants seen in NGS at the incidences shown (aligned upper sequences) are below the limit of detection of Sanger sequencing. Middle table is the computed summed proportions of AGCT corresponding with the Sanger chromatogram (below). Corresponds with Figure 6C.

(B) Analysis for non-mCherry positive other cells in *Tg(mpx:Cas9)* line, injected with *trim33*-gRNA. The variants seen in NGS at the incidences shown (aligned upper sequences) are below the limit of detection of Sanger sequencing. Middle table is the computed summed proportions of AGCT corresponding with the Sanger chromatogram (below). Corresponds with Figure 5C.

Disease Models & Mechanisms • Supplementary information

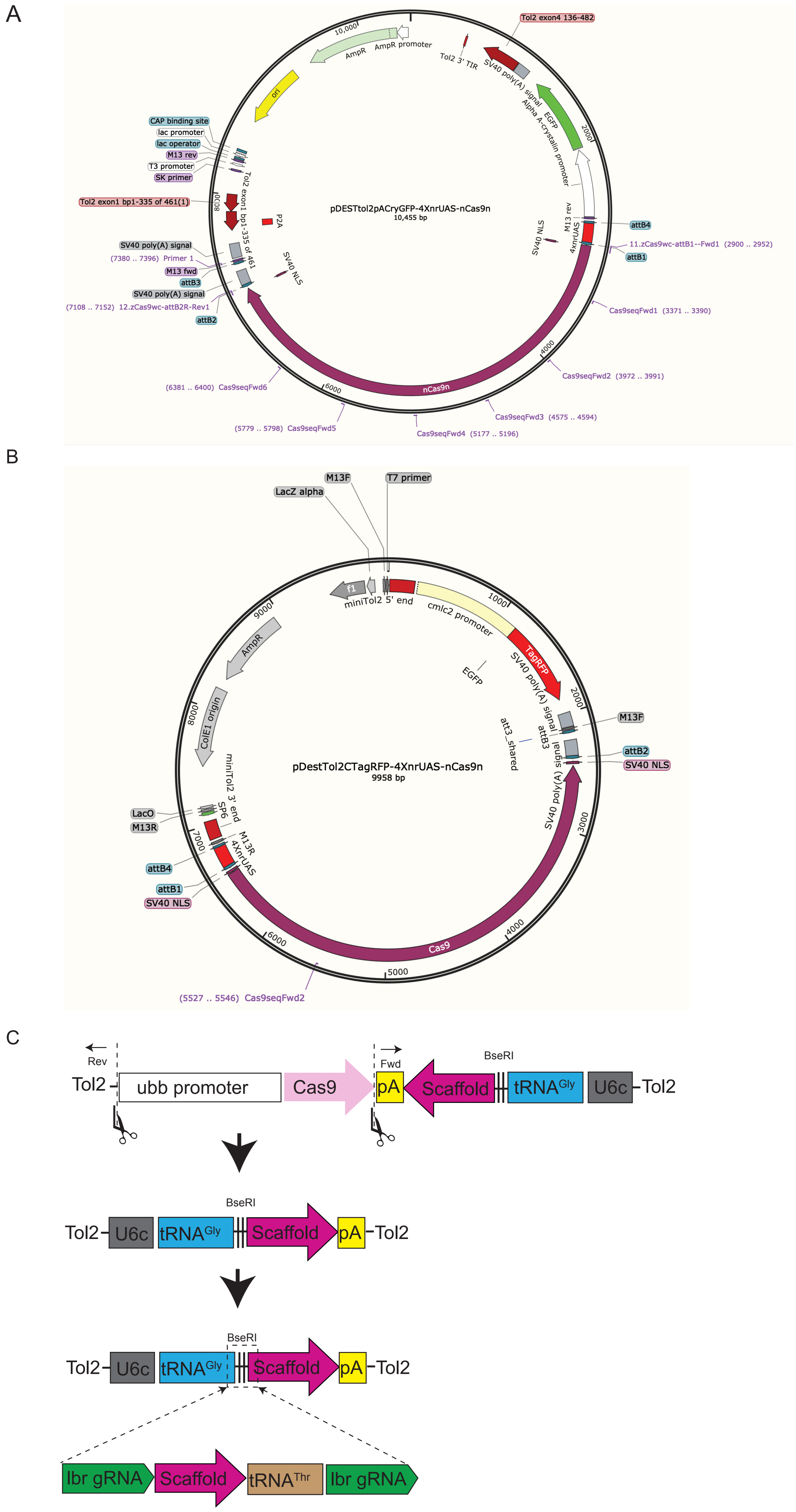

Figure S7. Plasmid maps of Cas9 and *lbr*-gRNA transgenes.

(A) Shows circular annotation of *4xUAS:NLS-Cas9, cmlc2:RFP* plasmid

(B) Shows circular annotation of *4xUAS:NLS-Cas9,cryaa:EGFP* plasmid

(C) Linear map annotation of steps for generating plasmid *lbr-gRNA*

Disease Models & Mechanisms • Supplementary information

Table S1. Primers

Name	Sequence (5'-3')	Purpose
Cas9_F1	GATCAGTGTAGCGTCCAGCA	PCR
Cas9_R1	GTGGAGCAGCACAAGCATTA	
Cas9_F2	TCTGGCTACCCAGCTCCTTA	RT- PCR,
Cas9_R2	GGCATTCTGCAGACAGTGAA	RT-qPCR
Cas9_F3	CTGGTATAGCGCCTTCTTGC	RT-PCR
Cas9_R3	CAGGCACAGCATCAAGAAAA	
lbrFwd	TTGCAGAGTGTGGTCATTTTTC	PCR, NGS*
lbrRev	ACACCCACTGCAAGTAAAACCT	
trim33gRNA1Fwd	CGACGACATCCAGCGATAG	PCR, NGS*
trim33gRNA1Rev	CGACATCTCGGTAACCACAG	
trim33gRNA2Fwd	TGAGCAGTCCAGAAGGAAGTT	PCR
trim33gRNA2Rev	TGCAGGAGGAAAGGTTATCG	
βactin_Fwd	GCTGACAGGATGCAGAAGGA	RT-PCR
βactin_Rev	TAGAAGCATTTGCGGTGGAC	
Ppial_Fwd	ACACTGAAACACGGAGGCAAAG	RT- qPCR
Ppial_Rev	CATCCACAACCTTCCCGAACAC	

*NGS CS tags, forward- acactgacgacatggttctaca, reverse- tacggtagcagagacttggtct

Table S2. gRNAs

Name	Sequence (5'-3')	Target gene
lbrgRNA	GAACGGGAGCGACTGCGACG	lbr
mCherrygRNA1	CGCTTCAAGGTGCACATGGA	mCherry
mCherrygRNA2	TGGCCCCCTGCCCTTCGCCT	mCherry
trim33gRNA1	CTGTCCGCTGGGGTGGACGG	trim33
trim33gRNA2	GCTCAGTCCAGGACATTTTA	trim33

Dataset 1. NGS variant data from *lbr* gene editing

Click here to download Dataset 1

Dataset 2. NGS variant data from *trim33* gene editing

Click here to download Dataset 2

Disease Models & Mechanisms • Supplementary information
